# Research on the Prediction of Coal Workers’ Pneumoconiosis Based on Easily Detectable Clinical Data: Machine Learning Model Development and Validation Study

**DOI:** 10.2196/80156

**Published:** 2026-02-13

**Authors:** Haiquan Li, Jiaqi Jia, Xu Shi, Yudie Dong, Songquan Wang, Yuming Cui, Wenlu Hang, Dekun Zhang

**Affiliations:** 1 School of Chemical Engineering & Technology, China University of Mining and Technology, Xuzhou, Jiangsu, China; 2 Department of Respiratory and Critical Care Medicine, Second Affiliated Hospital of Xuzhou Medical University, 32 Meijian Road, Xuzhou, Jiangsu, China, 86 13813477830; 3 School of Mechatronic Engineering, Jiangsu Normal University, Xuzhou, Jiangsu, China

**Keywords:** coal workers’ pneumoconiosis, disease prediction, machine learning, clinical data, job-type

## Abstract

**Background:**

Coal workers’ pneumoconiosis (CWP) is the most prevalent occupational disease that causes irreversible lung damage. Early prediction of CWP is the key to blocking the irreversible process of pulmonary fibrosis. The prediction of CWP based on imaging data and biomarker detection is constrained due to high cost and poor convenience.

**Objective:**

The study aimed to use easily detectable clinical data to construct a prediction model for CWP through machine learning (ML) methods.

**Methods:**

A prediction framework was established using a moderate-sized dataset and multidimensional clinical features, including occupational information, lung function parameters, and blood indicators. Six ML algorithms (light gradient boosting machine, random forest, extreme gradient boosting, categorical boosting, support vector machine, and logistic regression) were trained and evaluated using a stratified 5-fold cross-validation and a held-out test set. Hyperparameter optimization was performed using a unified Optuna-based strategy to ensure fair comparison across models. Model interpretability was assessed using Shapley Additive Explanation on top-performing models. In addition, an ablation analysis was conducted by retraining models after excluding job type to assess the independent predictive value of clinical biomarkers.

**Results:**

All 6 models achieved consistently high predictive performance, and the differences among the top-performing models were small on the test set. After Optuna-based optimization, light gradient boosting machine and categorical boosting achieved high test-set area under curve values (0.974 and 0.975, respectively), while extreme gradient boosting achieved the highest recall (0.926) and *F*_1_-score (0.952). Compared with the baseline models, hyperparameter optimization resulted in only minor performance changes, indicating robust prediction under the current feature set and evaluation protocol. Shapley Additive Explanation analysis consistently identified age, forced expiratory volume/forced vital capacity, and platelet count as key contributors to CWP risk prediction. The ablation analysis further showed that model performance remained strong after removing job type, supporting the independent predictive value of clinical features beyond occupational history.

**Conclusions:**

The research results have confirmed the potential of combining simple multidimensional features with ML algorithms for predicting CWP and provided new ideas for early diagnosis and intervention of patients with CWP.

## Introduction

As a traditional fossil energy source, coal has long held an important position in the global energy system. A large amount of respiratory coal dust can be generated during coal mining, processing, loading, and transportation and enter the human lungs through diffusion and sedimentation, inducing the occurrence of coal workers’ pneumoconiosis (CWP) [1]. The pathogenesis of CWP is complex, characterized by strong concealment in the early stage, high mortality rate in the later stage, and poor quality of life for patients. Once diagnosed, the course of the disease is irreversible and there is currently no effective cure in clinical practice [2,3]. Early identification of CWP can delay the deterioration of the condition and prevent it from developing into progressive mass fibrosis or respiratory failure.

High kilovoltage X-ray examination is the gold standard for CWP diagnosis. To avoid the problem of overlapping and occlusion of tissue and organ images, computed tomography detection technology has also been used for CWP diagnosis [4,5]. However, the imaging differences among early patients with CWP are not significant, and there are also issues such as high cost, high radiation risk, and convenient equipment use, which collectively constrain the early identification of patients with CWP. At present, the development of biomarker detection technology has significantly improved the clinical feasibility of CWP early screening [6,7]. As a measurable biological indicator, biomarkers can objectively reflect the physiological and pathological status of the body, such as proteins, genes, and metabolites, etc. The research on the expression levels of biomarkers in the serum of patients with CWP is the most extensive, including transforming growth factor-α [8], interleukin-8 [9], noncoding RNA (such as microRNA) [10], and common lipid metabolites such as phosphatidylethanolamines and free fatty acids [11], all of which have been proven to have important guiding significance for early identification of CWP. On the other hand, the occurrence and development of lung diseases usually have an impact on changes in lung microbiota and respiratory flora. MicroRNA expression profiles [12], surfactant-associated protein A and surfactant-associated protein D [13] in bronchoalveolar lavage fluid, and transforming growth factor-β, interleukin-1β, and matrix metalloproteinase-9 in sputum [14], as well as benzene and aldehydes in volatile organic compounds of exhaled breath [15], are also commonly used for early identification of CWP. However, metabolic processes are regulated by multiple factors. The lower specificity and sensitivity reduce the reliability of early screening of CWP through a single biomarker. Meanwhile, the high cost of detecting specific biomarkers also limits the early identification of patients with CWP.

Previous studies have shown that blood routine examination, as an economical, efficient, and easy-to-operate screening method in clinical practice, has important guiding significance for early identification and risk assessment of diseases, especially in mining areas where medical resources are relatively scarce [16,17]. CWP usually leads to lung infections or the occurrence of inflammatory diseases, which are often reflected in lung function, coagulation function, inflammatory markers, etc. This provides the possibility for early identification of patients with CWP [18]. At present, there is a relative lack of research on CWP prediction based on routine clinical blood data. This study aims to develop a low-cost CWP early screening tool based on machine learning (ML) models. By establishing a 3D feature space of occupational exposure history, lung function parameters, and routine blood indicators, and combining 6 algorithms including light gradient boosting machine (LightGBM), random forest (RF), extreme gradient boosting (XGBoost), categorical boosting (CatBoost), support vector machine (SVM), and logistic regression (LR) for comparative analysis of predictive performance. In addition, an Optuna-based hyperparameter optimization strategy was applied to tune the models under a unified evaluation protocol. Finally, the Shapley Additive Explanation (SHAP) method was used to interpret model predictions and analyze the contributions of key parameters such as lung function indicators and blood indicators. The high-precision and interpretable prediction model constructed can provide theoretical basis for early screening of CWP.

## Methods

### Ethical Considerations

This study was approved by the Second Affiliated Hospital of Xuzhou Medical University ([2024] 082701). Due to the retrospective nature of the study and the use of deidentified data, the requirement for informed consent was waived by the institutional review board. To ensure privacy and confidentiality, all personal identifiers, such as names and national identification numbers, were removed and replaced with unique study IDs before data analysis. No financial compensation was provided to the participants as the data were extracted from routine clinical and physical examination records. Furthermore, we confirm that no identifiable information or images of individual participants are included in this manuscript or its supplementary materials.

### Data Sources

Two hundred eighty-seven patients with CWP were admitted to a large tertiary hospital from June 28, 2022, to September 20, 2024. Dust-exposed workers undergoing annual occupational health examinations at the same hospital from 2022 to 2024 were considered as controls. Considering some workers attended examinations in multiple years, records were deduplicated using a unique personal identifier, and only the most recent examination record per worker was retained, yielding 2446 unique controls. These data were retrospectively extracted from the hospital’s electronic medical records system and physical examination database. All participants were male, aged between 22 and 90 years, and were employees of a certain mining group. The testing report included common demographic information, job types, and routine biochemical indicators.

The clinical test data of dust-exposed workers and patients with CWP were intersected, and 17 indicators were selected as candidate features. These indicators included job type, age, forced expiratory volume/forced vital capacity (FEV1/FVC), white blood cell count (WBC), absolute neutrophil count (ANC), absolute lymphocyte count (ALC), absolute monocyte count (AMC), absolute eosinophil count (AEC), red blood cell count, hemoglobin, platelet count (PLT), alanine aminotransferase (ALT), glucose, triglycerides, cholesterol, high-density lipoprotein, and low-density lipoprotein. The raw dataset initially comprised 36 job types. However, when categorized by disease status, a highly significant class imbalance was observed that the number of healthy individuals exposed to dust was approximately 8 times that of patients with CWP. This severe imbalance phenomenon can cause the model to lean toward the majority class during training, thereby reducing its ability to recognize diseased samples and affecting the model’s generalization performance [19]. Therefore, the original 36 job titles were first merged into 11 broader job categories based on similarity in work environment and job tasks. And then examined the distribution of CWP cases across these categories and found substantial imbalance (eg, only 1 CWP case among vehicle drivers vs 218 cases among mixed excavation and coal mining workers). To avoid unstable estimates driven by rare categories and to ensure adequate case representation for modeling, we restricted the analytic cohort to 5 job categories with sufficient CWP case counts, including mixed excavation and coal mining workers, excavation workers, coal miners, winch operators, and conveyor operators. After this restriction, the final dataset included 1085 dust-exposed healthy individuals and 271 participants with CWP.

### Data Preprocessing

After verification, it was found that FEV1/FVC and low-density lipoprotein had missing values, accounting for 3.68% (50/1356) and 1.48% (20/1356), respectively. In order to avoid the impact of missing values on subsequent analysis and model training, the *k*-nearest neighbor (KNN) algorithm was used to fill in the missing value variables. The KNN imputation was performed within the training data for each fold in cross-validation, ensuring that the test data remained unseen during preprocessing. The specific calculation method is shown in equation (1).


(1) $$d\left(x_{i},x_{j}\right)\sqrt{\sum_{k=1}^{p}{\left(x_{ik}-x_{jk}\right)}^{2}}$$

Among them, *x_i_* and *x_j_,* respectively, represent the feature vectors of 2 samples, and *x_ik_* and *x_jk_,* respectively, represent the *k*th feature of these 2 samples.

The categorical variable job type was processed using one-hot encoding, which converts each category into a binary feature column. Categories include mixed excavation and coal mining, excavation workers, winch operators, conveyor operators, and coal miners. The remaining 15 continuous feature variables were standardized, and each feature was transformed into a distribution with a mean 0 (SD 1) for the model to analyze. Standardization was performed only on the training data, with the same scaling applied to the validation and test sets. The calculation method for the standard score of each feature is shown in equation (2).


(2) $$Z=\frac{x-\mu }{\sigma }$$

Among them, *x* is a certain value of the feature in the original data, *µ* is the mean of the feature, and *σ* is the SD of the feature.

### Statistical Analysis and Feature Selection

Statistical analysis was conducted using SPSS Statistics (version 26.0; IBM Corp). The normality of quantitative data was tested using the *K*-*S* test, and the homogeneity of variance was tested using the Levene test. Data with normal distribution were represented by mean (SD), and intergroup comparison was tested using 2 independent samples *t* test. The data with nonnormal distribution were represented by median (P25-P75), and Mann-Whitney *U* test was used for intergroup comparison. Categorical variables were represented by the number of examples (%), and comparison between groups was conducted using the chi-square test. The difference was statistically significant with *P*<.05. The Least Absolute Shrinkage and Selection Operator (LASSO) algorithm, an embedded feature selection method, was used for regression analysis to identify key feature variables associated with CWP, thereby reducing model complexity and enhancing generalization capability. LASSO regression achieved feature selection by performing α regularization on coefficients, shrinking the coefficients of less important features to 0 [20]. To ensure no information leakage, LASSO feature selection was performed within each fold’s training data, and the same selected features were applied to the validation data within each fold. This method was combined with correlation analysis in filtering methods to comprehensively select features.

### Construction and Evaluation of ML Models

Six representative ML models including tree-based ensemble learning models (LightGBM, XGBoost, RF, and CatBoost) and traditional classification algorithms (LR and SVM) were used for constructing CWP prediction models. A brief overview of each model’s key characteristics and its relevance to this study is provided below.

For tree-based ensemble learning models, XGBoost uses second-order Taylor expansion for high accuracy and speed, incorporates regularization to prevent overfitting, and supports parallel computing for efficient training [21]. LightGBM uses a leaf-wise growth strategy and histogram-based feature discretization for efficiency, with built-in class weight adjustments beneficial for imbalanced datasets [22]. CatBoost uses an ordered boosting strategy for better generalization, directly handles categorical features, and uses a symmetric tree structure to reduce overfitting [23]. RF builds multiple decision trees from bootstrapped samples, randomly selects features at each split, and aggregates predictions through voting for robust classification [24].

To compare model performance on imbalanced datasets, 2 traditional models were also selected for comparison with the ensemble models. LR as a generalized linear model predicts probabilities using a sigmoid function [25]. SVM finds an optimal hyperplane to separate classes, using slack variables and a radial basis function kernel for inseparable data. Class weights were also incorporated into its objective function for imbalance handling [26]. In this study, class weights were applied in the loss functions of both traditional models to effectively handle the class imbalance issue.

Python (version 3.8.0; Python Software Foundation) software was used for model training and evaluation, randomly dividing the dataset into training and test set in an 8:2 ratio. To further assess model stability, 5-fold stratified cross-validation within the training data was used. In this procedure, the dataset was randomly divided into 5 nonoverlapping subsets. In each fold, 4 subsets were used as the training set, and the remaining 1 subset was used for validation. This process was repeated 5 times, and the average performance across all folds was taken as the evaluation metric. The test set was only used for the final model evaluation, ensuring it remained unseen during model training and hyperparameter tuning. In the process of model training and evaluation, based on the confusion matrix, the performance of the model was comprehensively judged through accuracy, precision, recall, *F*_1_-score value, and the area under curve (AUC) of the subjects. The corresponding calculation formulas are as follows:


(3) $$Accuracy=\frac{TN+FN}{TP+FP}$$

(4) $$Precision=\frac{TP}{TP+FP}$$

(5) $$Recall=\frac{TP}{TP+FN}$$

(6) $$F1=\frac{2^{*}precision^{*}recall}{precision+recall}$$

**Figure 1.**
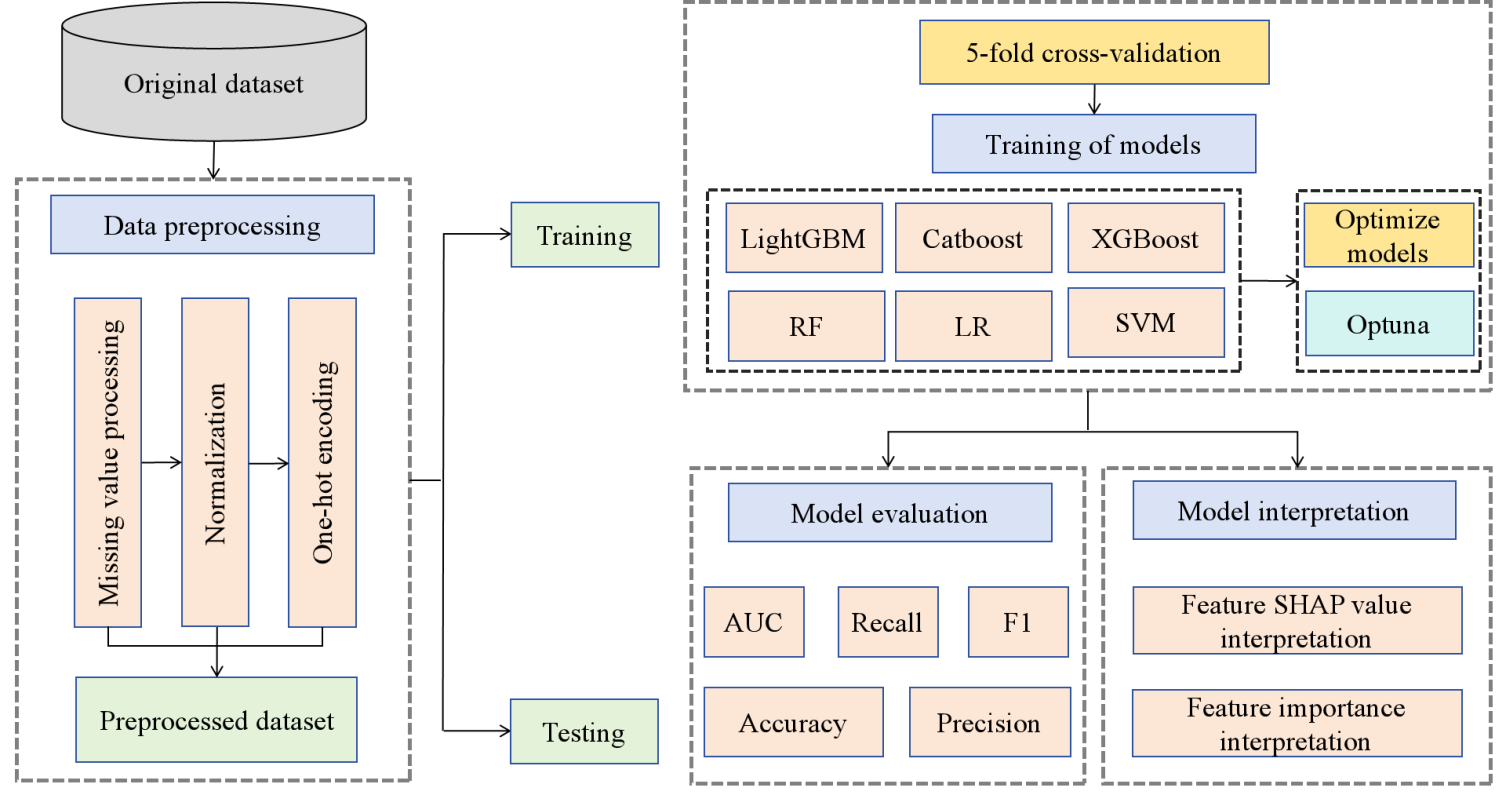
The technical roadmap of this study. AUC: area under the curve; CatBoost: categorical boosting; LightGBM: light gradient boosting machine; LR: logistic regression; RF: random forest; SHAP: Shapley Additive Explanation; SVM: support vector machine; XGBoost: extreme gradient boosting.

Among them, *TP* represents true positive, *TN* represents true negative, *FP* represents false positive, and *FN* represents false negative. Figure 1 shows the technical roadmap of this study.

## Results

### Results of Statistical Analysis and Feature Selection

Table 1 shows the comparison of basic characteristics between the dust-exposed workers and the patients with CWP. It is found that 13 indicators, including job type, age, FEV1/FVC, WBC, ANC, ALC, AMC, AEC, hemoglobin, PLT, ALT, cholesterol, and glucose, have statistical significance (*P*<.05) compared between the 2 groups.

**Table 1.** Comparison of basic characteristics between the dust-exposed workers and the patients with coal workers’ pneumoconiosis.

	Healthy	Disease	Test	Statistic	*P* value
Job type/Case	1085	271	Pearson χ^2^	—^a^	<.001
Mixed workers for excavation and coal mining, n (%)	160 (14.7)	219 (80.8)	—	—	—
Excavator workers, n (%)	253 (23.3)	35 (12.9)	—	—	—
Winch operator, n (%)	260 (24)	3 (1.1)	—	—	—
Conveyor operator, n (%)	234 (21.6)	6 (2.2)	—	—	—
Coal miners, n (%)	178 (16.4)	8 (3)	—	—	—
Age, median (IQR)	48 (39-52)	69 (62-77)	Mann-Whitney *U*	*U*=11,900, *Z*=−23.43	<.001
FEV1/FVC^b^ (%), median (IQR)	90 (86-97)	75.86 (68-89)	Mann-Whitney *U*	*U*=177,046, *Z*=12.02	<.001
WBC^c^ (×10^9^ /L), median (IQR)	6.36 (5.42-7.60)	5.84 (4.92-6.96)	Mann-Whitney *U*	*U*=177,670, *Z*=5.32	<.001
ANC^d^ (×10^9^ /L), median (IQR)	3.53 (2.86-4.39)	2.97 (2.21-3.98)	Mann-Whitney *U*	*U*=185,504, *Z*=6.67	<.001
ALC^e^ (×10^9^ /L), median (IQR)	2.21 (1.82-2.69)	2.01 (1.58-2.72)	Mann-Whitney *U*	*U*=163,138, *Z*=2.80	.005
AMC^f^ (×10^9^ /L), median (IQR)	0.39 (0.33-0.48)	0.47 (0.38-0.68)	Mann-Whitney *U*	*U*=94,124, *Z*=−9.17	<.001
AEC^g^ (×10^9^ /L), median (IQR)	0.16 (0.10-0.25)	0.13 (0.07-0.19)	Mann-Whitney *U*	*U*=174,854, *Z*=4.83	<.001
RBC^h^ (×10^12^ /L), median (IQR)	4.89 (4.64-5.12)	4.85 (4.46-5.33)	Mann–Whitney *U*	*U*=150,982, *Z*=0.69	.49
HB^i^ (g/L), median (IQR)	151 (144-158)	148 (136-160)	Mann-Whitney *U*	*U*=161,103, *Z*=2.44	.01
PLT^j^ (×10^9^ /L), median (IQR)	244 (211-276)	200 (158.50-240)	Welch t	*U*=208,796, *Z*=10.71	<.001
ALT^k^ (U/L), median (IQR)	20 (15-27)	17 (12-24)	Mann-Whitney *U*	*U*=180,185, *Z*=5.75	<.001
GLU^l^ (mmol/L), median (IQR)	5.25 (4.85-5.75)	4.91 (4.41-5.62)	Mann-Whitney *U*	*U*=180,125, *Z*=6.14	<.001
TG^m^ (mmol/L), median (IQR)	1.42 (0.99-2.33)	1.28 (0.96-2.01)	Mann-Whitney *U*	*U*=157,897, *Z*=1.89	.06
CHOL^n^ (mmol/L), median (IQR)	4.86 (4.29-5.52)	4.48 (3.67-5.22)	Mann-Whitney *U*	*U*=181,528, *Z*=5.98	<.001
HDL^o^ (mmol/L), median (IQR)	1.27 (1.12-1.47)	1.23 (1.05-1.54)	Mann-Whitney *U*	*U*=150,479, *Z*=0.60	.55
LDL^p^ (mmol/L), median (IQR)	2.67 (2.29-3.11)	2.69 (2.13-3.21)	Mann-Whitney *U*	*U*=129,398, *Z*=0.67	.50

a Not available.

b FEV1/FVC: forced expiratory volume/forced vital capacity.

c WBC: white blood cell.

d ANC: absolute neutrophil count.

e ALC: absolute lymphocyte count.

f AMC: absolute monocyte count.

g AEC: absolute eosinophil count.

h RBC: red blood cell count.

i HB: hemoglobin.

j PLT: platelet count.

k ALT: alanine aminotransferase.

l GLU: glucose.

m TG: triglycerides.

n CHOL: cholesterol.

o HDL: high-density lipoprotein.

p LDL: low-density lipoprotein.

Figure S1 in Multimedia Appendix 1 showed the cross-validation curve of LASSO regression. When the α value is low, the model may contain too many irrelevant features, resulting in significant errors (overfitting). When the α value is large, the model may remove too many important features, which also leads to an increase in error (underfitting). At the optimal α value, the cross-validation error is minimized. The LASSO coefficient plot was shown in Figure S2 in Multimedia Appendix 1, which showed that the coefficients of 9 features, including job-type mixed excavation coal, job-type excavation worker, job-type conveyor operator, job-type winch operator, age, FEV1/FVC, AMC, PLT, and ANC, were not 0 at the optimal α value. This indicated the criticality of these features and their significant explanatory power for the target variable; therefore, they should be retained in the final model for prediction.

**Figure 2.**
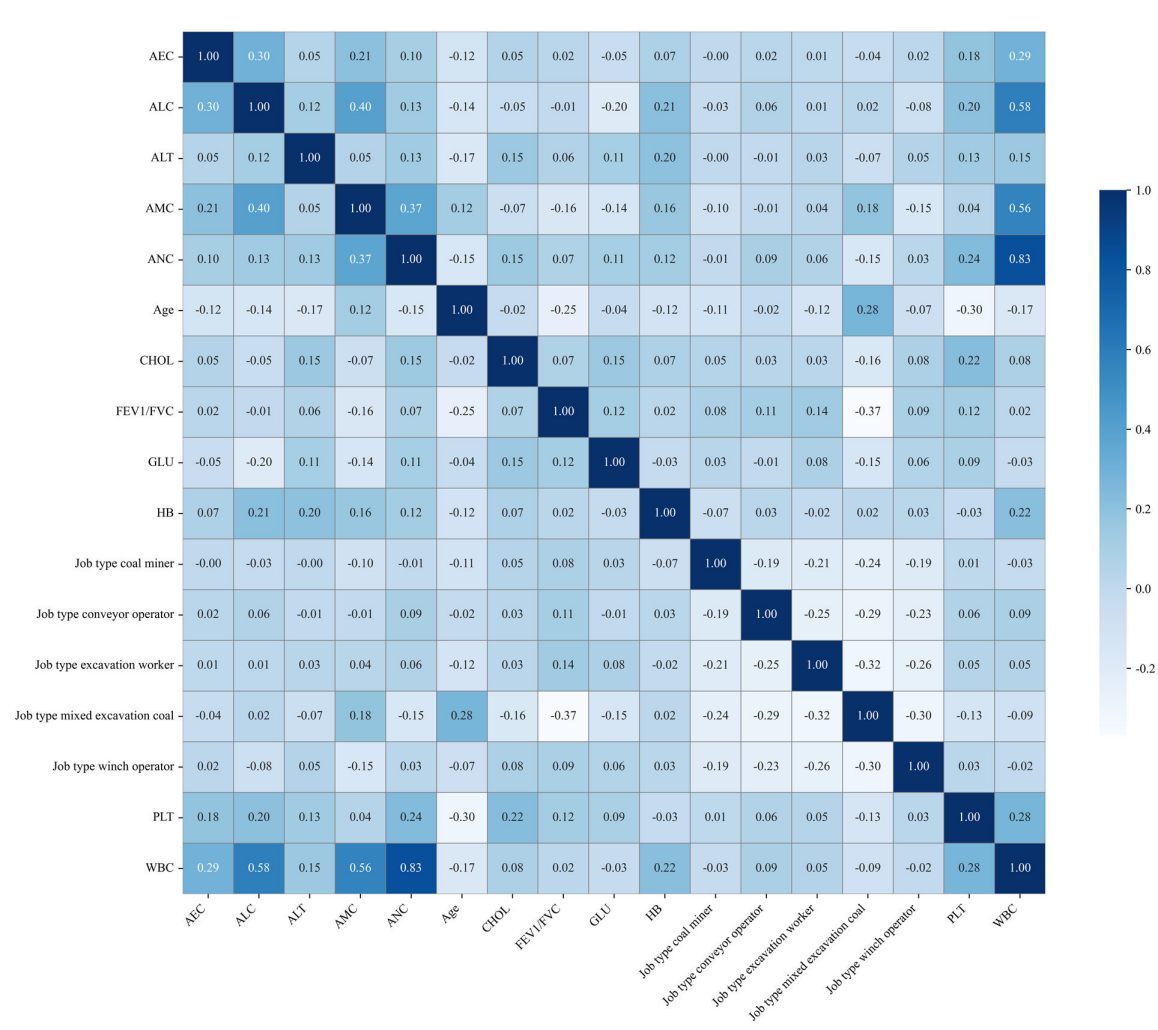
Spearman correlation coefficient matrix. AEC: absolute eosinophil count; ALC: absolute lymphocyte count; ALT: alanine aminotransferase; AMC: absolute monocyte count; ANC: absolute neutrophil count; CHOL: cholesterol; FEV1/FVC: forced expiratory volume/forced vital capacity; GLU: glucose; HB: hemoglobin; PLT: platelet count; WBC: white blood cell count.

In order to avoid the problem of multicollinearity caused by strong correlation between features, this study used correlation analysis in a filtering method to comprehensively select features based on the 17 features selected through statistical analysis and LASSO regression screening in the early stage. By calculating the Spearman correlation coefficient matrix (Figure 2), the threshold was set to an absolute value of *r* greater than 0.8, and highly correlated terms in the feature pairs were selected. Based on the absolute value of LASSO regression weights, the features that contribute more to the target were retained, thereby eliminating redundant variables. Specifically, a Spearman correlation matrix was computed on the training data, and pairs with an absolute value of *r* greater than 0.8 were considered highly correlated. For each highly correlated pair, the feature with the larger absolute LASSO coefficient was retained and the other feature was removed. In the current dataset, WBC and ANC showed high correlation (*r*=0.833); thus, WBC was removed and ANC was retained. After redundancy filtering, 16 nonredundant features were used as inputs for subsequent model development, including job-type mixed excavation coal, job-type excavation worker, job-type conveyor operator, job-type winch operator, job-type coal miner, age, FEV1/FVC, ANC, ALC, AMC, AEC, hemoglobin, PLT, ALT, cholesterol, and glucose.

### Evaluation of the CWP Prediction Model

The selected 16 clinical features were input as feature variables into 6 ML models, and 5-fold cross-validation was performed for each model during training. The performance of each model was comprehensively evaluated based on the test-set data, and the output results of each model were organized and are summarized in Table 2. Meanwhile, the visualized results of the data in Table 2 are shown in Figure S3 in Multimedia Appendix 1. From the figure, it can be seen that the AUC of each fold of the 6 models in cross-validation was consistently high (all folds >0.90), indicating that the generalization ability of the 6 models is strong and stable. The AUC values of the 6 models were ranked in descending order as CatBoost (0.979), LightGBM (0.978), XGBoost (0.976), RF (0.972), SVM (0.968), and LR (0.967). This result indicated that the performance of ensemble learning models on imbalanced datasets was superior to traditional models, verifying the advantages of ensemble learning models in dealing with such problems [27]. Taking into account accuracy, precision, recall, and *F*_1_-score, both the LightGBM and RF models achieved the highest accuracy (0.982), precision (1), and *F*_1_-score (0.951), while the LR model had the highest recall (0.926). Therefore, based on the overall performance across multiple evaluation indicators, LightGBM and RF were preliminarily considered as top-performing predictive models.

**Table 2.** Evaluation of predictive performance of different models^b^.

Model	Accuracy	Precision	Recall	*F*_1_-score	AUC^a^
LightGBM^c^	0.982^b^	1.000^b^	0.907	0.951^b^	0.978
CatBoost^d^	0.978	0.980	0.907	0.942	0.979^b^
XGBoost^e^	0.978	0.980	0.907	0.942	0.976
RF^f^	0.982^b^	1.000^b^	0.907	0.951^b^	0.972
LR^g^	0.956	0.862	0.926^b^	0.893	0.967
SVM^h^	0.963	0.907	0.907	0.907	0.968

a AUC: area under the curve.

b Significant values.

c LightGBM: light gradient boosting machine.

d CatBoost: categorical boosting.

e XGBoost: extreme gradient boosting.

f RF: random forest.

g LR: logistic regression.

h SVM: support vector machine.

In order to further improve the predictive performance and to ensure a fair comparison among candidate models, this study used the Optuna algorithm to optimize the hyperparameters of all 6 ML models (LightGBM, CatBoost, XGBoost, RF, LR, and SVM) under the same optimization budget, and also conducted 5-fold cross-validation during training. The output results of each optimized model were summarized in Table 3, and the visualized results were shown in Figure S4 in Multimedia Appendix 1. The results showed that the AUC of each fold in cross-validation was consistently high (all folds >0.90), indicating that the generalization ability of the 6 models was strong and stable. After applying an equivalent hyperparameter optimization strategy, the overall performance of the 6 models remains high and the differences among the top-performing models were small. Specifically, CatBoost and LightGBM achieved high test-set AUC values (0.975 and 0.974, respectively). In addition, XGBoost achieved the highest recall (0.926) and *F*_1_-score (0.952) on the test set. Compared with the baseline results, hyperparameter optimization led to only small changes in performance. Overall, the 6 models maintained consistently high performance under the current evaluation protocol.

**Table 3.** Performance evaluation of optimized models.

Model	Accuracy	Precision	Recall	*F*_1_-score	AUC^a^
LightGBM^b^-Optuna	0.982	1	0.907	0.951	0.974
CatBoost^c^-Optuna	0.982	1	0.907	0.951	0.975
XGBoost^d^-Optuna	0.982	0.98	0.926	0.952	0.969
RF^e^-Optuna	0.974	0.961	0.907	0.933	0.968
LR^f^-Optuna	0.952	0.847	0.926	0.885	0.968
SVM^g^-Optuna	0.967	0.925	0.907	0.916	0.962

a AUC: area under the curve.

b LightGBM: light gradient boosting machine.

c CatBoost: categorical boosting.

d XGBoost: extreme gradient boosting.

e RF: random forest.

f LR: logistic regression.

g SVM: support vector machine.

### Model Interpretability

In order to gain a deeper understanding of the impact of various clinical features on the model’s prediction results, this study used the SHAP method to conduct interpretability analysis on the 2 representative top-performing models LightGBM-Optuna and CatBoost-Optuna. The calculation method was shown in Equation 7.


(7) $$SHAP(y)=\;SHAP(base)+\sum_{i=1}^{n}SHAP(x_{i})$$

Among them, *SHAP (base*) is the baseline value of the entire model, and *SHAP (x_i_*) is the contribution of each sample to the final prediction result.

**Figure 3.**
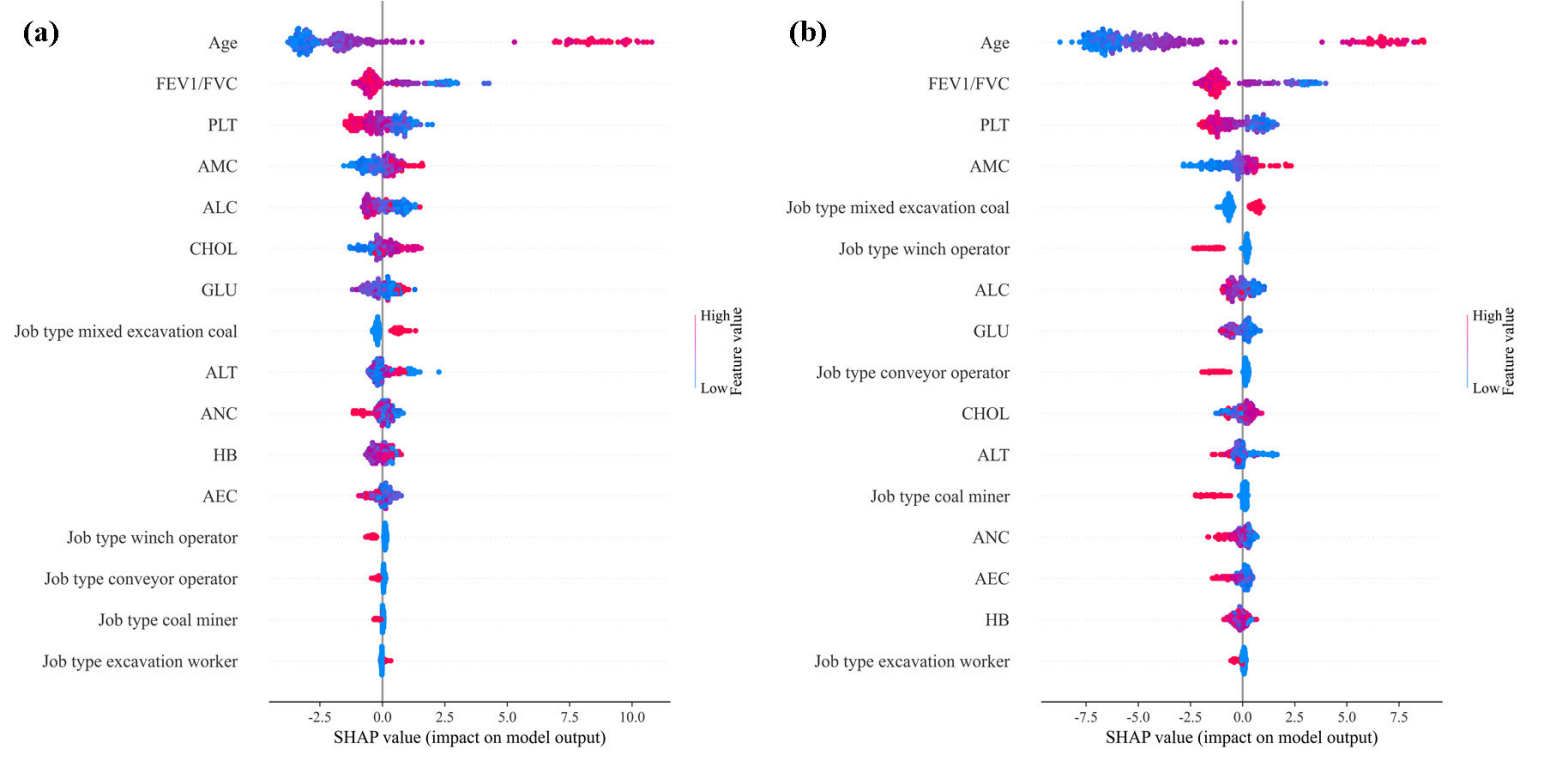
Summary chart of SHAP values. (a) LightGBM-Optuna; (b) CatBoost-Optuna. AEC: absolute eosinophil count; ALC: absolute lymphocyte count; ALT: alanine aminotransferase; AMC: absolute monocyte count; ANC: absolute neutrophil count; CHOL: cholesterol; FEV1/FVC: forced expiratory volume/forced vital capacity; GLU: glucose; HB: hemoglobin; PLT: platelet count; SHAP: Shapley Additive Explanations.

The summary results of SHAP values are shown in Figure 3, which displayed the distribution of SHAP values for 17 input feature variables. Each point in the figure represented a feature, and the position of the point represented the SHAP value of the feature, which was the contribution of the feature to the model output. If the SHAP value is positive, it indicates that the feature increases the risk of disease and has a positive impact on the output results. Conversely, if it is negative, it indicates that the feature reduces the risk of disease and has a negative impact on the output results. In addition, the color range from blue to red reflects the actual value of the feature, with red indicating high values and blue indicating low values. The darker the color, the stronger the impact of the feature on the target variable. Overall, both models showed consistent patterns in feature effects. Age is the most influential variable, and higher age values were mainly associated with positive SHAP values, suggesting that older individuals tended to have a higher predicted disease risk. In contrast, higher values of FEV1/FVC were mostly distributed on the negative side, indicating that better lung function (higher FEV1/FVC) was related to a lower predicted risk. PLT showed a similar tendency, with higher values generally corresponding to negative SHAP values. On the other hand, higher AMC values tended to correspond to positive SHAP values, indicating a positive association with increased predicted risk. These results suggested that the model predictions were largely driven by age-related factors and lung function indicators, together with selected hematological and biochemical variables.

**Figure 4.**
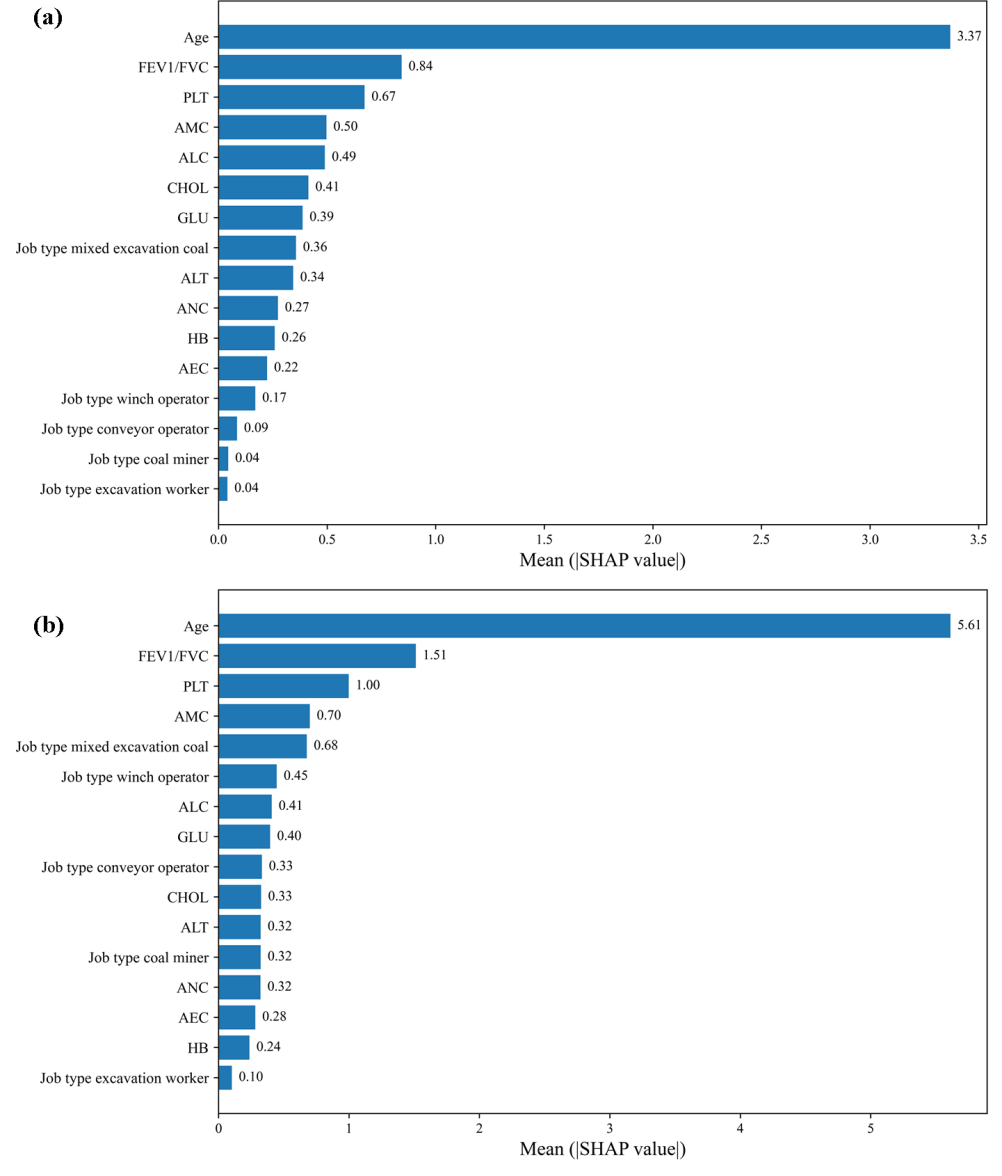
Matrix diagram of SHAP feature importance. (a) LightGBM-Optuna; (b) CatBoost-Optuna. AEC: absolute eosinophil count; ALC: absolute lymphocyte count; ALT: alanine aminotransferase; AMC: absolute monocyte count; ANC: absolute neutrophil count; CHOL: cholesterol; FEV1/FVC: forced expiratory volume/forced vital capacity; GLU: glucose; HB: hemoglobin; PLT: platelet count; SHAP: Shapley Additive Explanation.

Figure 4 showed the SHAP feature importance matrix, which arranged the average SHAP absolute value of each feature from high to low. The horizontal axis represented the contribution value, and the larger the value, the greater the contribution to the model results. In both the Optuna-tuned LightGBM and CatBoost models, age showed the highest contribution, followed by FEV1/FVC and PLT, which indicates that these variables play the most important roles in the prediction of CWP risk.

To further examine how the top 3 influential features affect the model output, SHAP dependence plots were generated (Figure S5 in Multimedia Appendix 1). As shown in the dependence plot for age, the SHAP values generally increase with age, and the risk contribution became more pronounced after approximately 55‐60 years, suggesting that older age was associated with a higher predicted disease risk. For FEV1/FVC, lower values correspond to higher SHAP values, indicating an increased risk, whereas higher values (approximately 85%‐100%) were associated with SHAP values close to zero or negative, suggesting a lower predicted risk. Similarly, the PLT dependence plot showed that lower PLT levels tended to contribute positively to disease risk, while higher PLT values (approximately 250‐350×10^9^/L) were more often associated with negative SHAP values, indicating a reduced predicted risk.

## Discussion

### Principal Findings

In clinical research, it is very common for medical records to have missing values in a certain examination due to the complexity of data collection and individual differences among different patients. By calculating the Euclidean distance between samples, KNN interpolation can identify *K*-neighboring samples that are most similar to missing values and then use the average feature information of neighboring samples to fill in the missing values, effectively restoring the integrity of the data [28]. As a categorical variable, the values of job type do not have a sequential relationship, and categories with larger values do not necessarily have greater weights than categories with smaller values. In order to enable ML models to better capture the relationship between feature variables and target variables, single hot encoding was applied to categorical features in this study [29]. The remaining 15 characteristic variables are all continuous features, but their units and ranges of values vary greatly. For example, PLT is measured in unit ×10^9/L and has a wide range of variable values, while glucose is measured in unit mmol/L and has a smaller range of values. This inconsistent scale may lead to the model being more sensitive to certain features with larger numerical ranges during training and ignoring other features with smaller scales, thereby affecting the training effectiveness of the model. Standardizing irregular continuous variables is often the key to solving such problems [30].

Feature selection is a crucial step in ML applications, aimed at selecting the most relevant features to the target variable in order to improve model performance and interpretability [31]. The common feature selection methods mainly include embedded, wrapped, and filtered methods [32]. As an embedded feature selection method, LASSO uses regression analysis to screen out key feature variables related to CWP, reducing model complexity and improving model generalization ability. In this study, LASSO regression analysis is applied to compress the coefficients of some unimportant features to 0 by performing α regularization on the coefficients, thereby achieving feature selection [20]. The preprocessed dataset still suffers from class imbalance, with the number of dust-exposed workers without CWP being about 4 times that of patients with CWP. Regarding the issue of class imbalance, commonly used methods in model construction include data sampling and ensemble learning [33]. In order to preserve the distribution characteristics of the original data as much as possible and avoid the bias and noise that may be introduced by data oversampling methods [34], ensemble learning models were used to handle imbalanced data in this study. Specifically, we selected decision tree-based ensemble learning models such as LightGBM, RF, CatBoost, and XGBoost and compared them with traditional LR and SVM models.

To ensure a fair comparison among candidate models, this study adopted a unified hyperparameter optimization strategy based on Optuna. Optuna is a Bayesian optimization framework that uses a tree-structured Parzen estimator to efficiently explore the hyperparameter space by prioritizing promising regions. Under the same optimization budget and the same stratified *k*-fold cross-validation protocol, all 6 models were tuned and evaluated consistently. The results show that after optimization, all models achieved consistently high cross-validated performance, while the differences among the top-performing models remained small on the held-out test set. This finding suggests that the current feature set and evaluation setting already provide strong predictive ability, and further improvements are more likely to depend on feature refinement or decision strategy rather than extensive hyperparameter tuning. In addition, the top-performing models show comparable overall performance, but each presents advantages under different evaluation priorities. Specifically, models such as LightGBM and CatBoost demonstrate stronger overall discrimination, whereas XGBoost tends to perform better when recall- or *F*_1_-related sensitivity is emphasized. Therefore, LightGBM and CatBoost were both retained as top-performing models for subsequent interpretability analysis. Job type reflects different dust exposure scenarios in coal mining and therefore contributes to CWP risk prediction, which is consistent with epidemiological evidence [35]. The concentration, particle size, and composition of coal dust have a significant impact on the pathogenesis and prevalence of CWP [36,37]. The different working scenarios in coal mines in the same region also have a significant impact on the prevalence of CWP. The excavator workers are mainly responsible for developing tunnels, and the cut rocks are rich in free silica. The pathogenicity of silica dust is much higher than that of coal dust, which can lead to more severe pulmonary fibrosis (silicosis) and a shorter onset period [38,39]. Coal miners mainly come into contact with coal dust (carbon-based dust), which has relatively weaker pathogenicity compared to silica dust and slower disease progression. At the same time, the excavation face is a temporary work site, and the ventilation and dust removal facilities are usually not as complete as those in the coal mining face, resulting in greater difficulty in dust control [40]. Mixed workers for excavation and coal mining are exposed to silica dust and coal dust simultaneously, and the synergistic effect of the 2 types of dust may accelerate lung damage [41,42].

To examine whether the clinical variables provide predictive value beyond occupational history, we conducted an ablation analysis by retraining the baseline models after removing the job-type variable. Because hyperparameter tuning led to only minor changes in performance, using baseline models for this analysis was sufficient to evaluate the independent contribution of clinical variables. The results of each model are summarized in Table 4, which showed that model performance remained highly robust. For example, the AUC of LightGBM only slightly changed from 0.978 (with job type) to 0.973 (without job type), indicating that the physiological signals captured by clinical features and biomarkers are major contributors to the model’s predictive capability.

**Table 4.** Ablation analysis of model performance without job type.

Model	Accuracy	Precision	Recall	*F*_1_-score	AUC^a^
LightGBM^b^	0.982	1	0.907	0.951	0.973
CatBoost^c^	0.978	0.98	0.907	0.942	0.977
XGBoost^d^	0.982	1	0.907	0.951	0.978
RF^e^	0.982	1	0.907	0.951	0.976
LR^f^	0.963	0.893	0.926	0.909	0.969
SVM^g^	0.974	0.98	0.889	0.932	0.971

a AUC: area under curve.

b LightGBM: light gradient boosting machine.

c CatBoost: categorical boosting.

d XGBoost: extreme gradient boosting.

e RF: random forest.

f LR: logistic regression.

g SVM: support vector machine.

This finding is also supported by the SHAP interpretation results. The decrease in FEV1/FVC reflects impaired lung function, while the corresponding increase in SHAP value suggests that ventilation function may be an important feature of CWP. This is consistent with the results of existing studies suggesting that alveolar-arterial oxygen gradient in lung function can be used as a predictor of CWP [43]. Platelets, as important cells for hemostasis and coagulation, play a role by participating in systemic inflammatory and immune responses, providing new therapeutic targets for inflammatory diseases [44]. For example, a previous study found that lower PLT levels were associated with a higher risk of developing severe *mycoplasma pneumoniae* pneumonia [45]. Due to the important role of platelets in inflammation and tissue repair, this phenomenon may be related to inflammation or weakened immune function leading to lung damage. These findings emphasize the importance of clinical features in CWP risk assessment and provide new perspectives for a deeper understanding of the pathogenesis of CWP.

Despite its contributions, this study has several limitations. First, the cohort was derived from a single center and a specific occupational group, which may introduce regional or selection bias and limit generalizability to other settings. Second, although model performance was evaluated rigorously, interpretability remains limited and warrants further investigation. Most importantly, smoking history was not available in the retrospective physical examination records. Because smoking is a major confounder for both lung function and inflammatory biomarkers, part of the observed discrimination may reflect unmeasured differences in smoking behavior rather than CWP status alone. Furthermore, differences in physical demands and lifestyle factors associated with distinct job roles could potentially influence certain biomarkers. While our analysis indicates strong independent predictive value for the biomarkers, future studies should consider more granular lifestyle adjustments.

### Conclusions

This study developed a ML-based model for CWP prediction using multidimensional clinical features. The 6 candidate models achieved consistently high performance, and Optuna-based tuning resulted in only small changes, suggesting robust prediction under the current protocol. SHAP analysis identified age, FEV1/FVC, and PLT as key contributors to CWP risk prediction. Moreover, ablation analysis showed that the models remained highly accurate even without job type, indicating that clinical biomarkers provide strong predictive signals beyond occupational information. These results support the potential of routine clinical data for early CWP screening and intervention.

## Supplementary material

10.2196/80156 Multimedia Appendix 1 Cross-validation curve of least absolute shrinkage and selection operator regression, least absolute shrinkage and selection operator coefficient chart, cross-validation of 6 models and receiver operating characteristic curves of the final model, receiver operating characteristic curves of optimized model, and Shapley Additive Explanation dependency graph.

## Abbreviations

AEC: absolute eosinophil count
ALC: absolute lymphocyte count
ALT: alanine aminotransferase
AMC: absolute monocyte count
ANC: absolute neutrophil count
AUC: area under curve
CatBoost: categorical boosting
CWP: coal workers’ pneumoconiosis
FEV1/FVC: forced expiratory volume/forced vital capacity
KNN: k-nearest neighbor
LASSO: Least Absolute Shrinkage and Selection Operator
LightGBM: light gradient boosting machine
LR: logistic regression
ML: machine learning
PLT: platelet count
RF: random forest
SHAP: Shapley Additive Explanation
SVM: support vector machine
WBC: white blood cell count
XGBoost: extreme gradient boosting
