# Supplementary material for: Research on the Prediction of Coal Workers’ Pneumoconiosis Based on Easily Detectable Clinical Data: Machine Learning Model Development and Validation Study
Source: JMIR Med Inform. 2026 Feb 13;14:e80156. doi: 10.2196/80156 (PMC12904349; doi:10.2196/80156)
Supplement: Multimedia Appendix 1 [file medinform-v14-e80156-s001.docx]

**Figure S1.** Cross-Validation curve of Lasso regression. This curve displays the mean cross-validation error (MSE) with standard deviation as Alpha varies. The optimal Alpha value corresponding to the minimum mean MSE, is explicitly marked to indicate the selected regularization strength for feature selection.


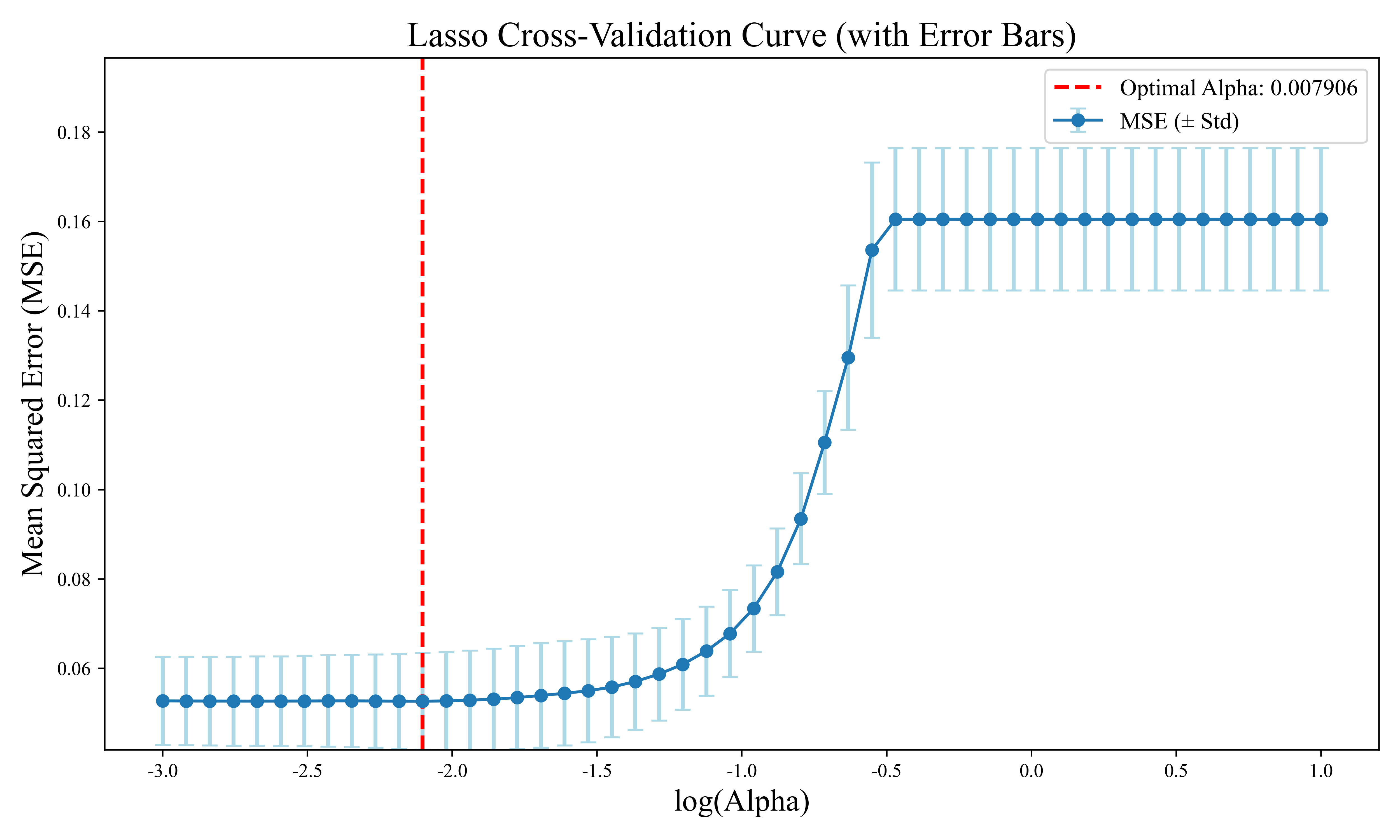


**Figure S2.** Lasso coefficient chart. This plot displays the change in coefficients for each feature as the Alpha value varies. A vertical line is drawn at the optimal Alpha value identified from Figure S1. Features whose coefficients remain non-zero at this optimal Alpha value are selected as the most relevant features for the prediction model. FEV1/FVC: forced expiratory volume/forced vital capacity; WBC: white blood cell count; ANC: absolute neutrophil count; ALC: absolute lymphocyte count; AMC: absolute monocyte count; AEC: absolute eosinophil count; RBC: red blood cell count; HB: hemoglobin; PLT: platelet count; ALT: alanine aminotransferase; GLU: glucose; TG: triglycerides; CHOL: cholesterol; HDL: high-density lipoprotein.


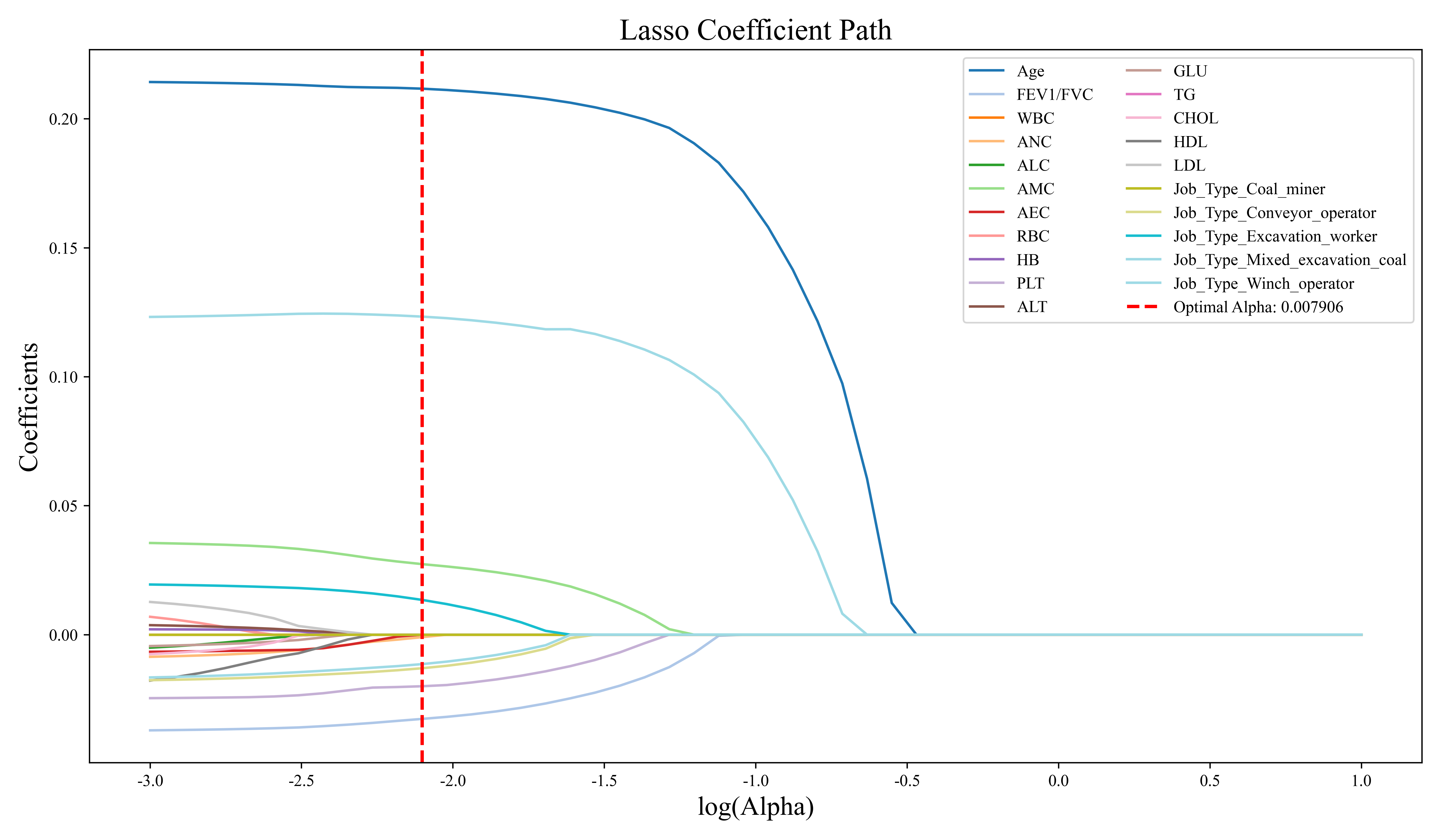


**Figure S3.** Cross-Validation of 6 models and ROC curves of the final model (A) LightGBM, (B) Catboost, (C) XGBOOST, (D) RF, (E) LR, (F) SVM. LightGBM: Light Gradient Boosting Machine; CatBoost: Categorical Boosting; XGBoost: eXtreme Gradient Boosting; RF: Random Forest; LR: Logistic Regression; SVM: Support Vector Machine.


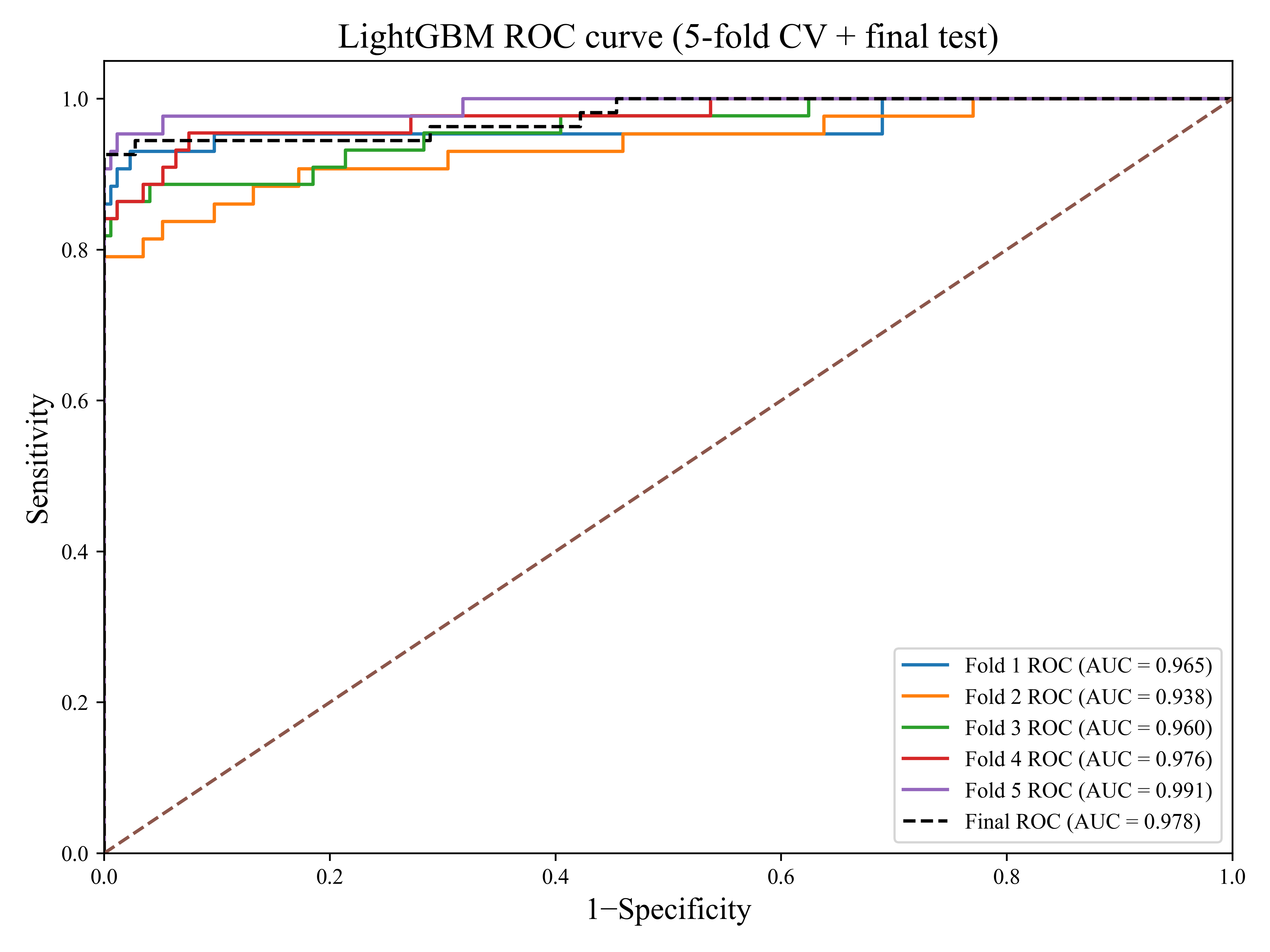


**(a)**


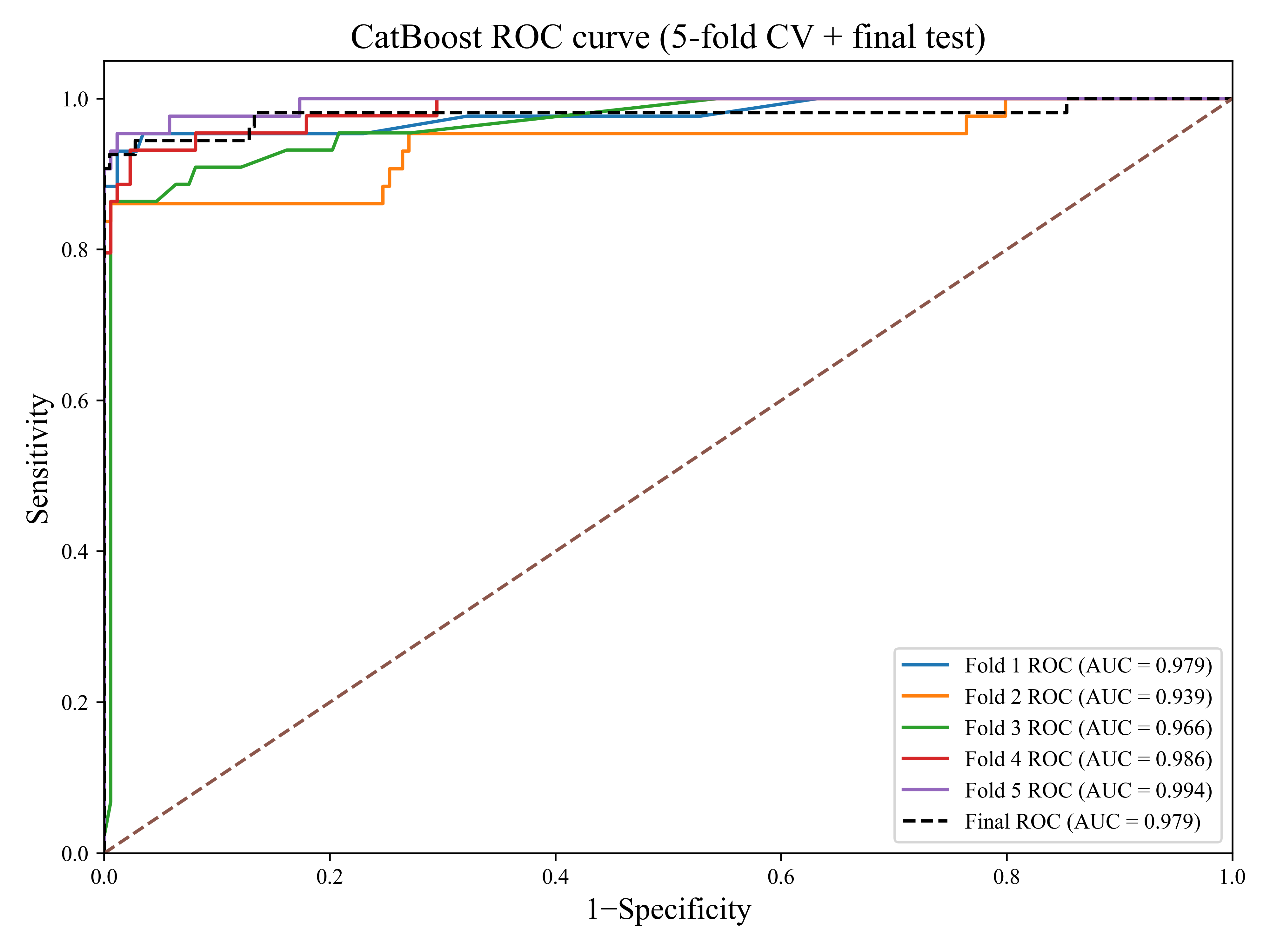


**(b)**


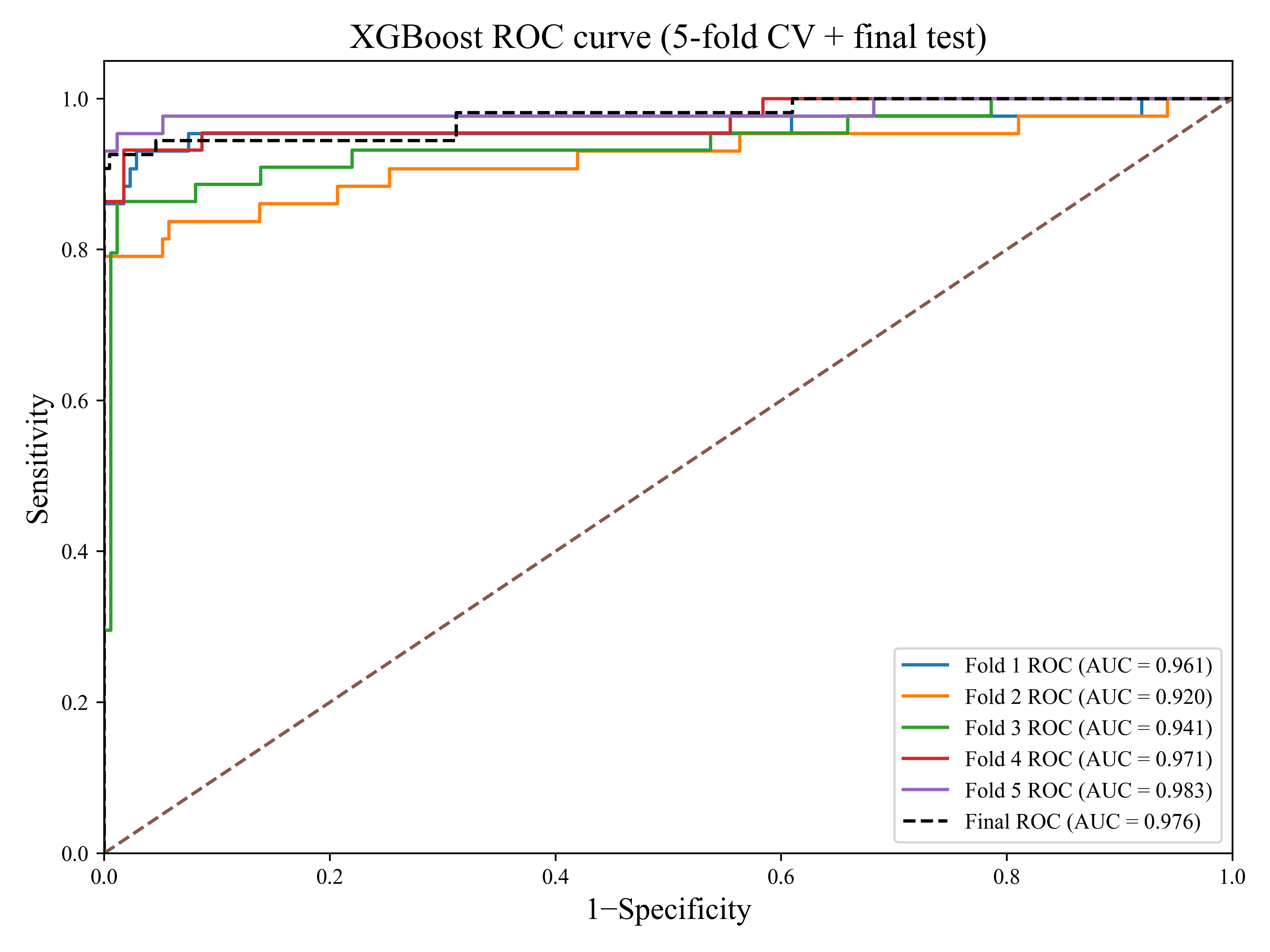


**(c)**


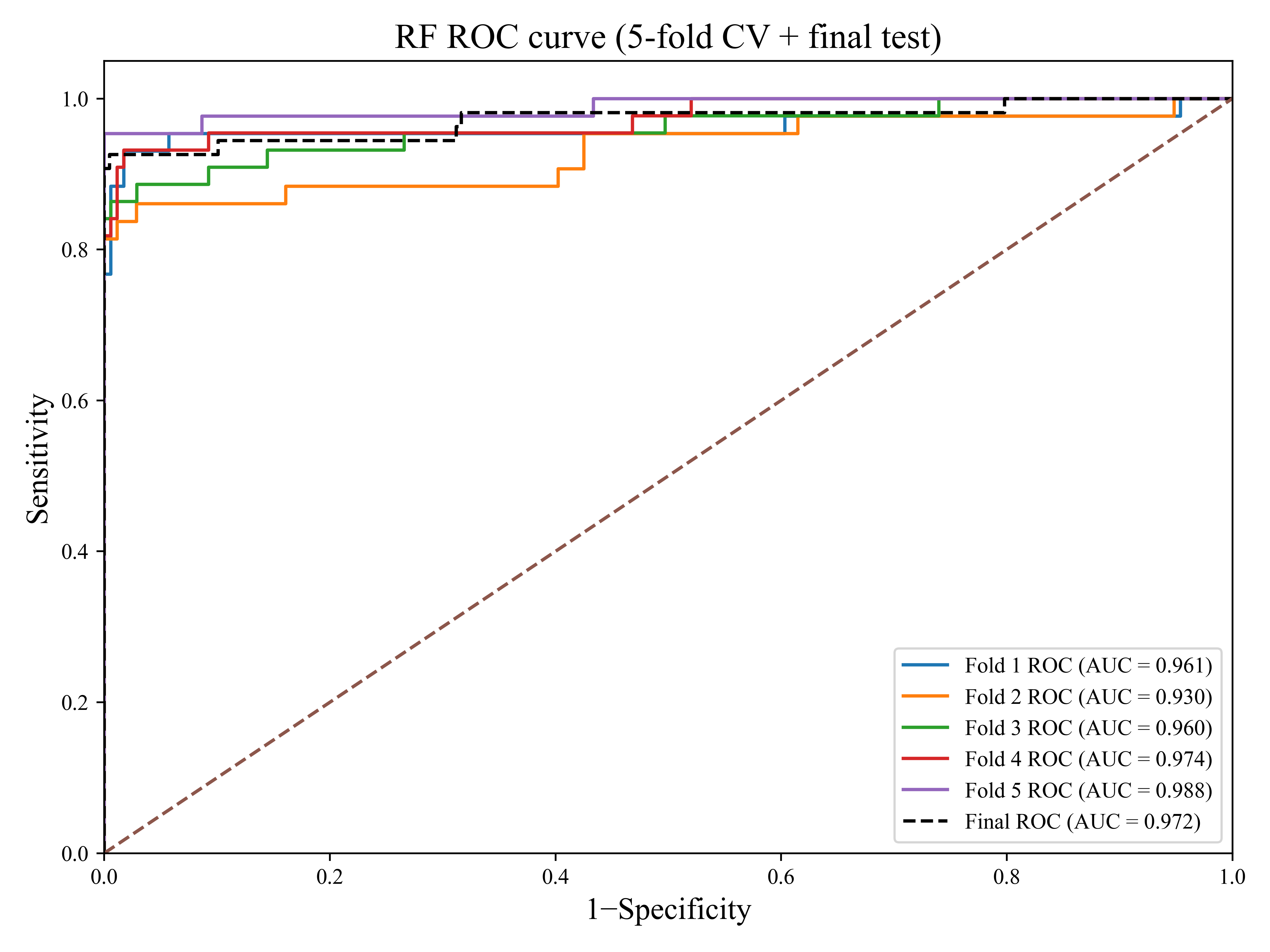


**(d)**


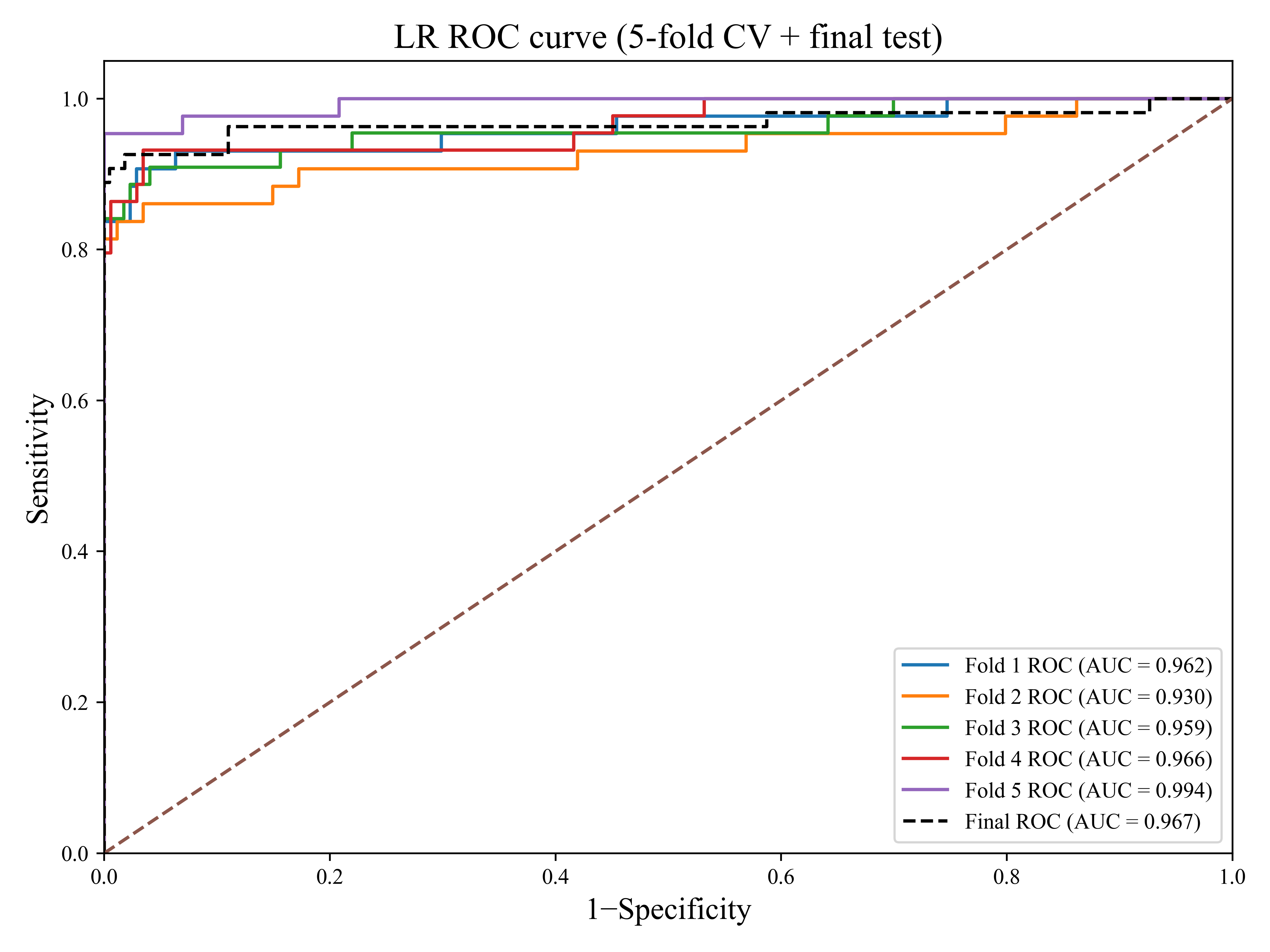


**(e)**


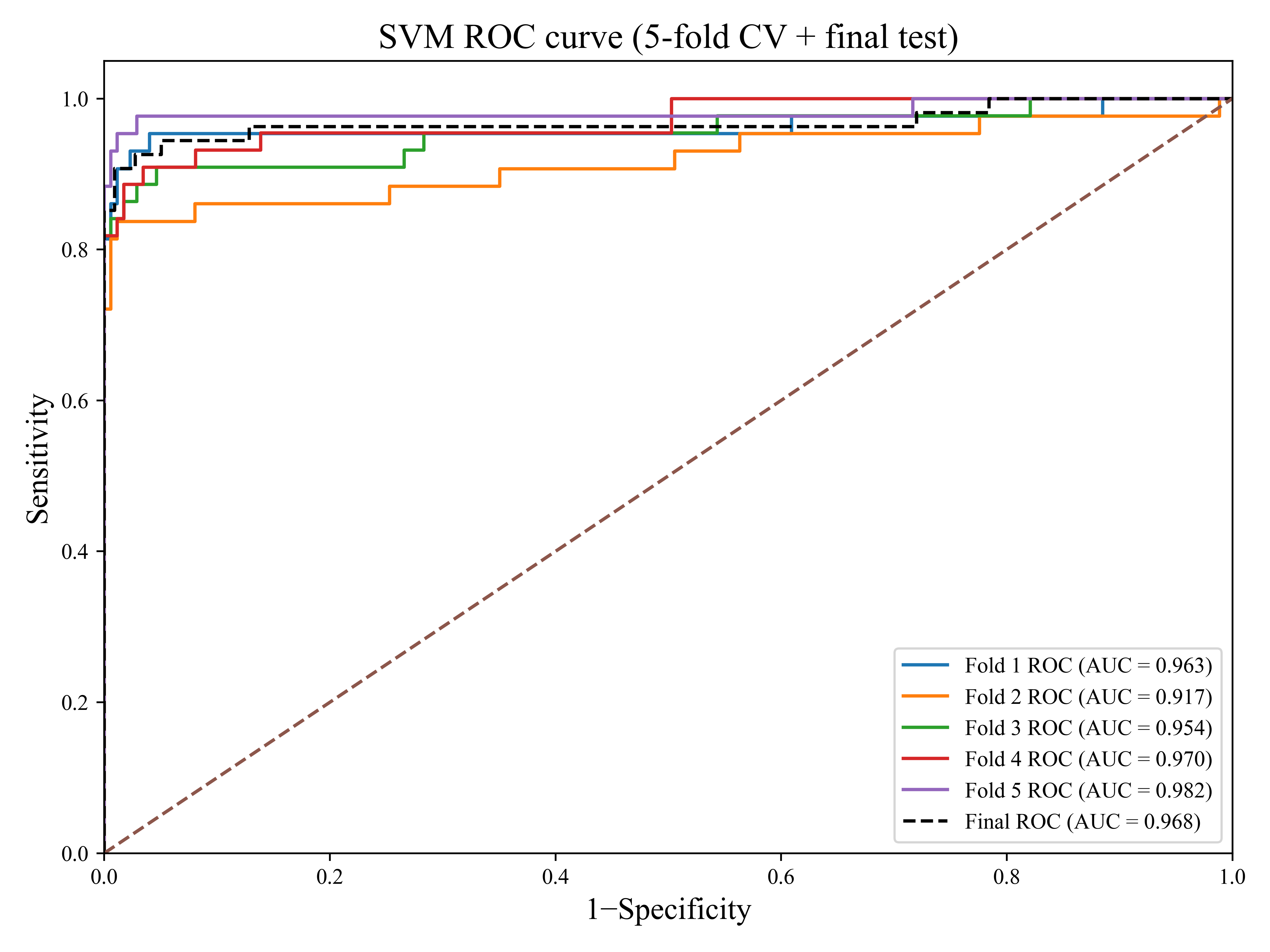


**(f)**

**Figure S4.** ROC curves of optimized model (a) LightGBM-Optuna, (b) Catboost-Optuna, (c) XGBOOST-Optuna, (d) RF-Optuna, (e) LR-Optuna, (f) SVM-Optuna. LightGBM: Light Gradient Boosting Machine; CatBoost: Categorical Boosting; XGBoost: eXtreme Gradient Boosting; RF: Random Forest; LR: Logistic Regression; SVM: Support Vector Machine.


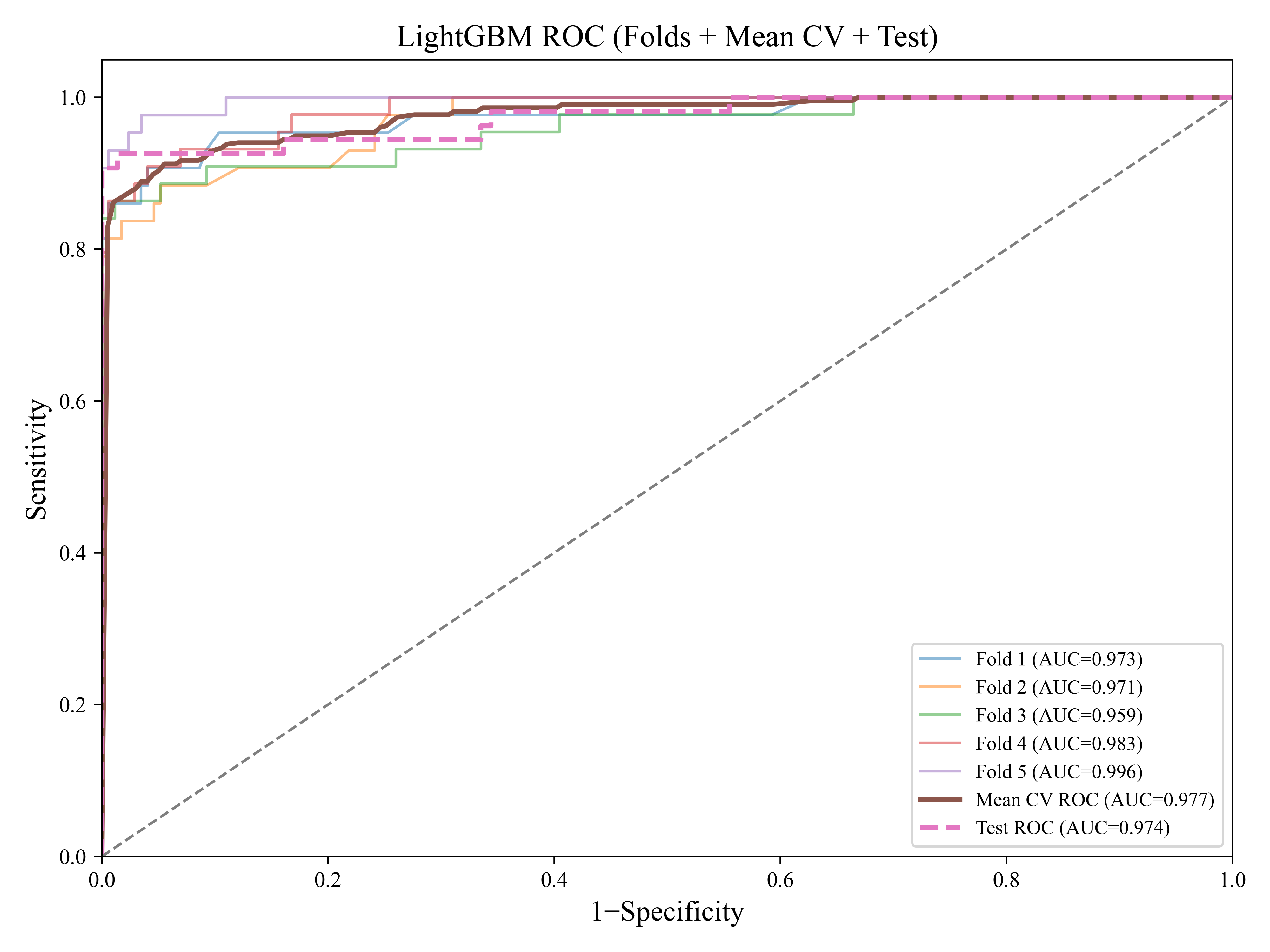


**(a)**


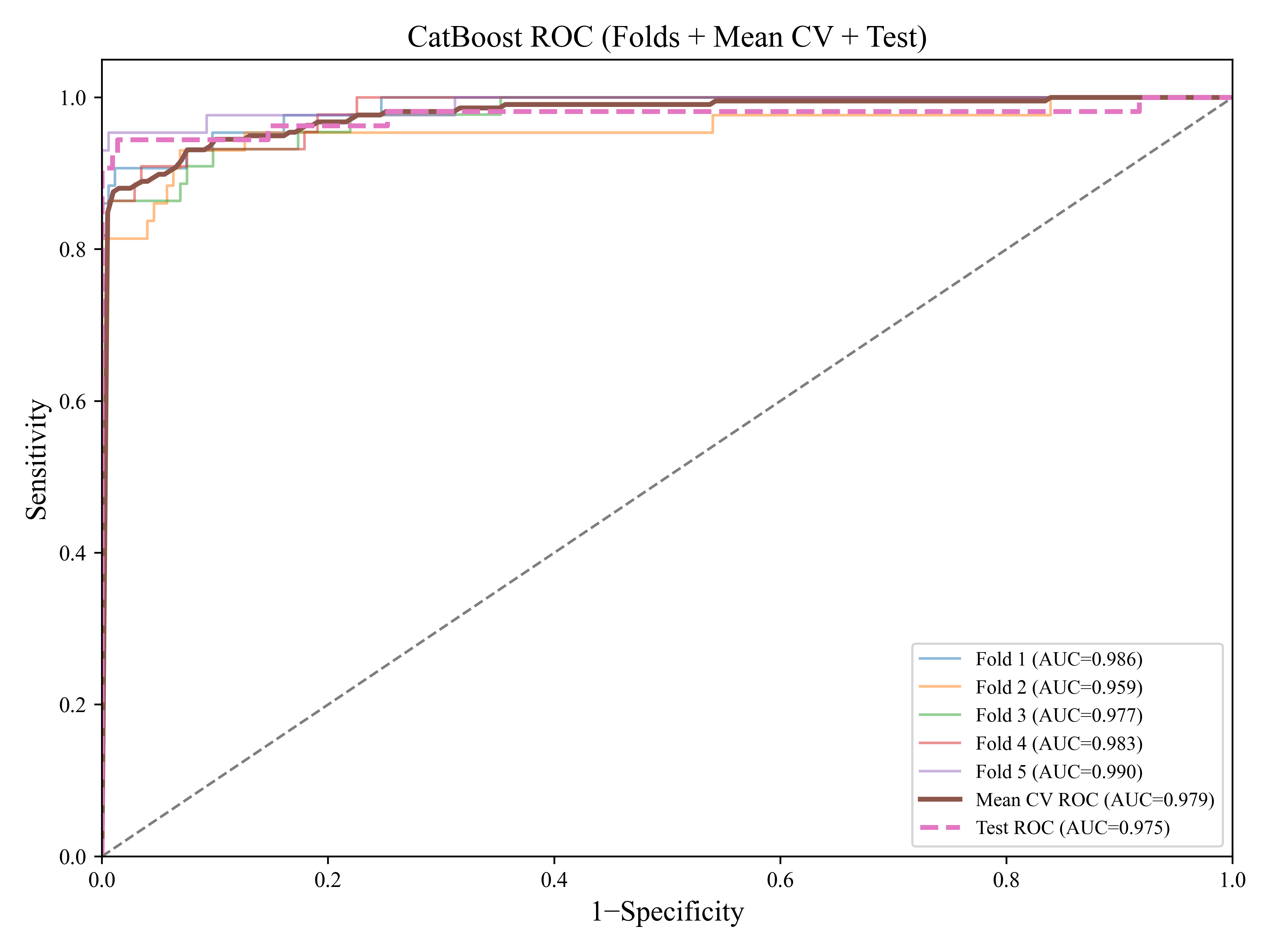


**(b)**


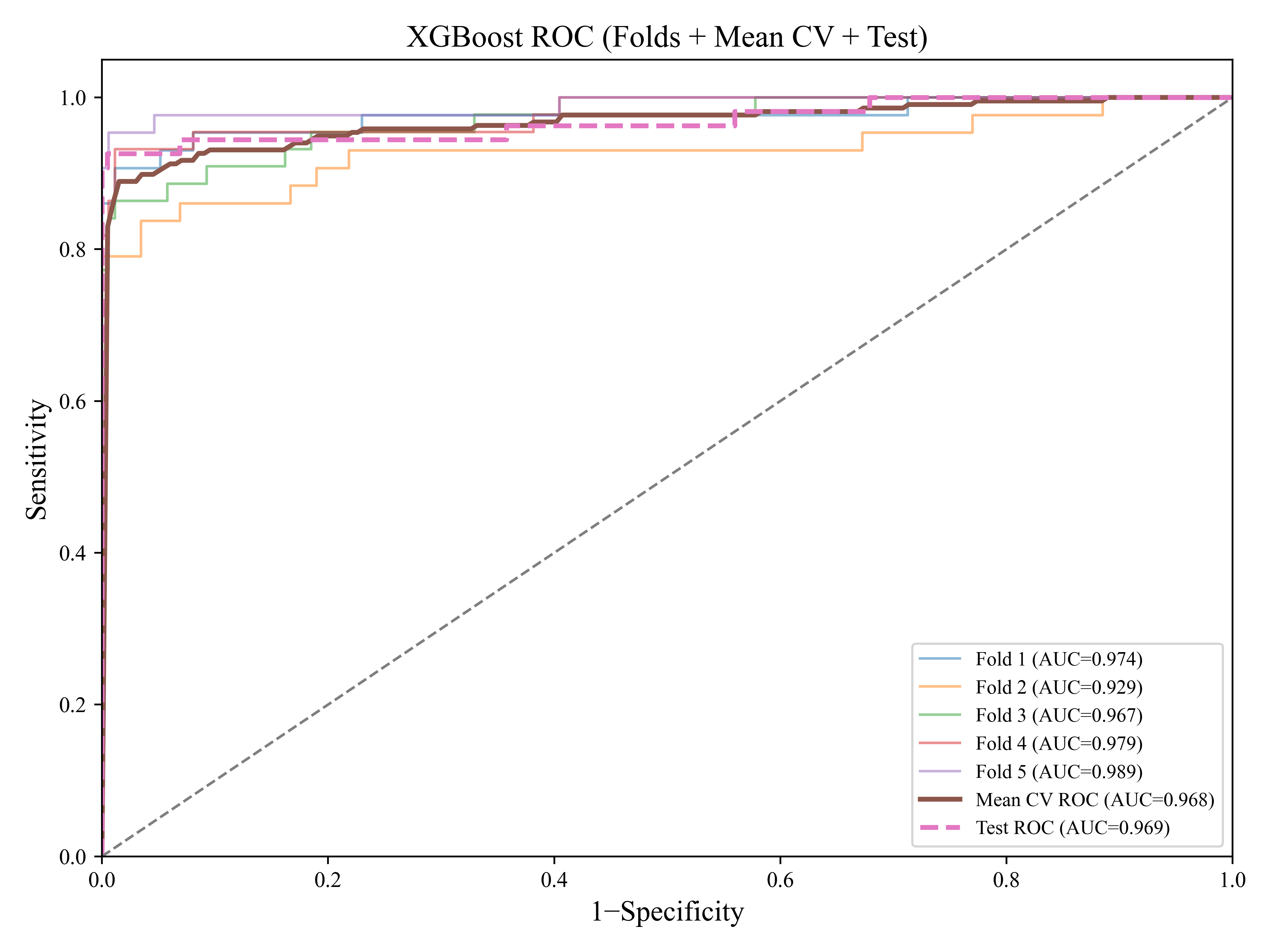


**(c)**


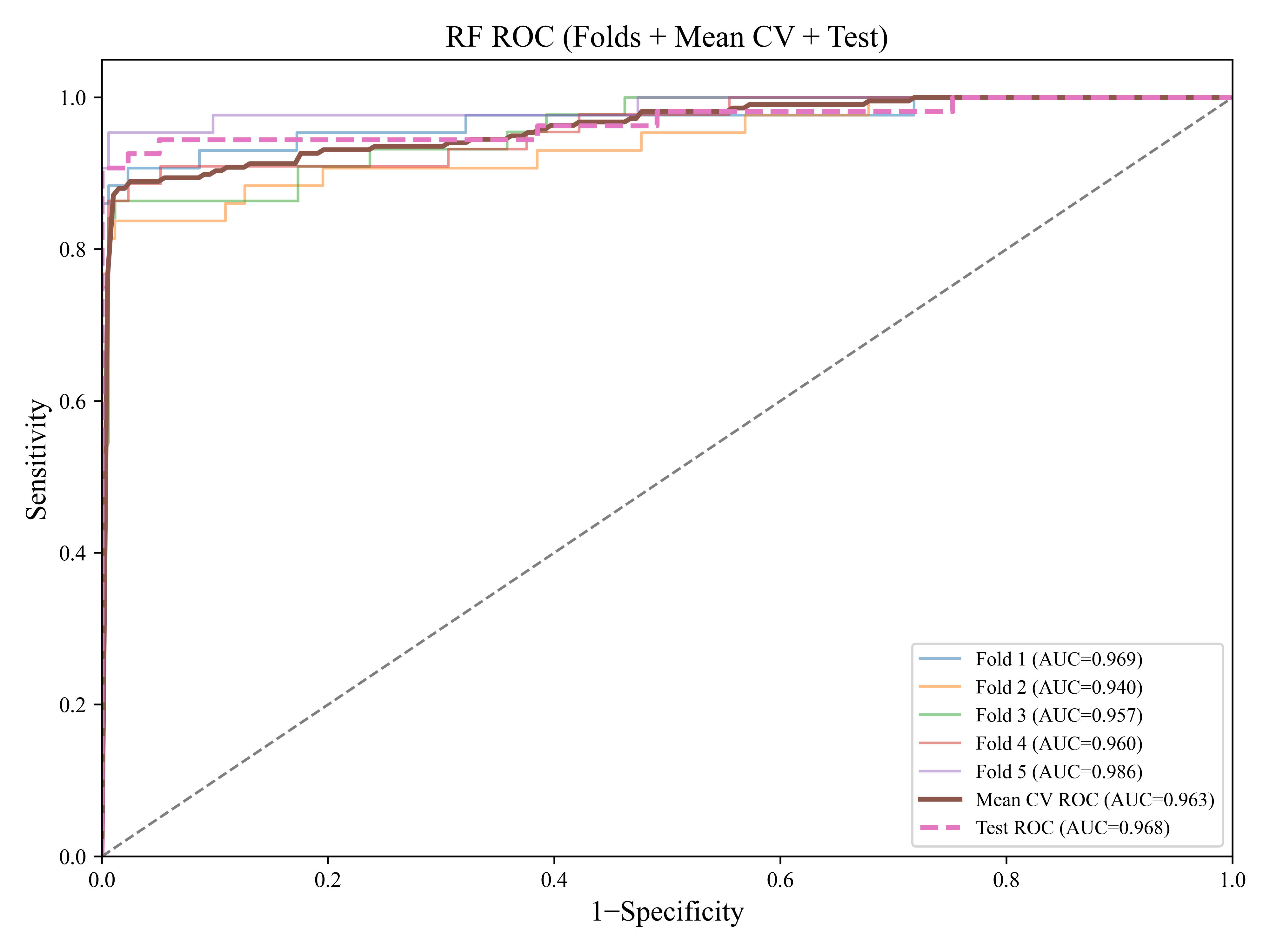


**(d)**


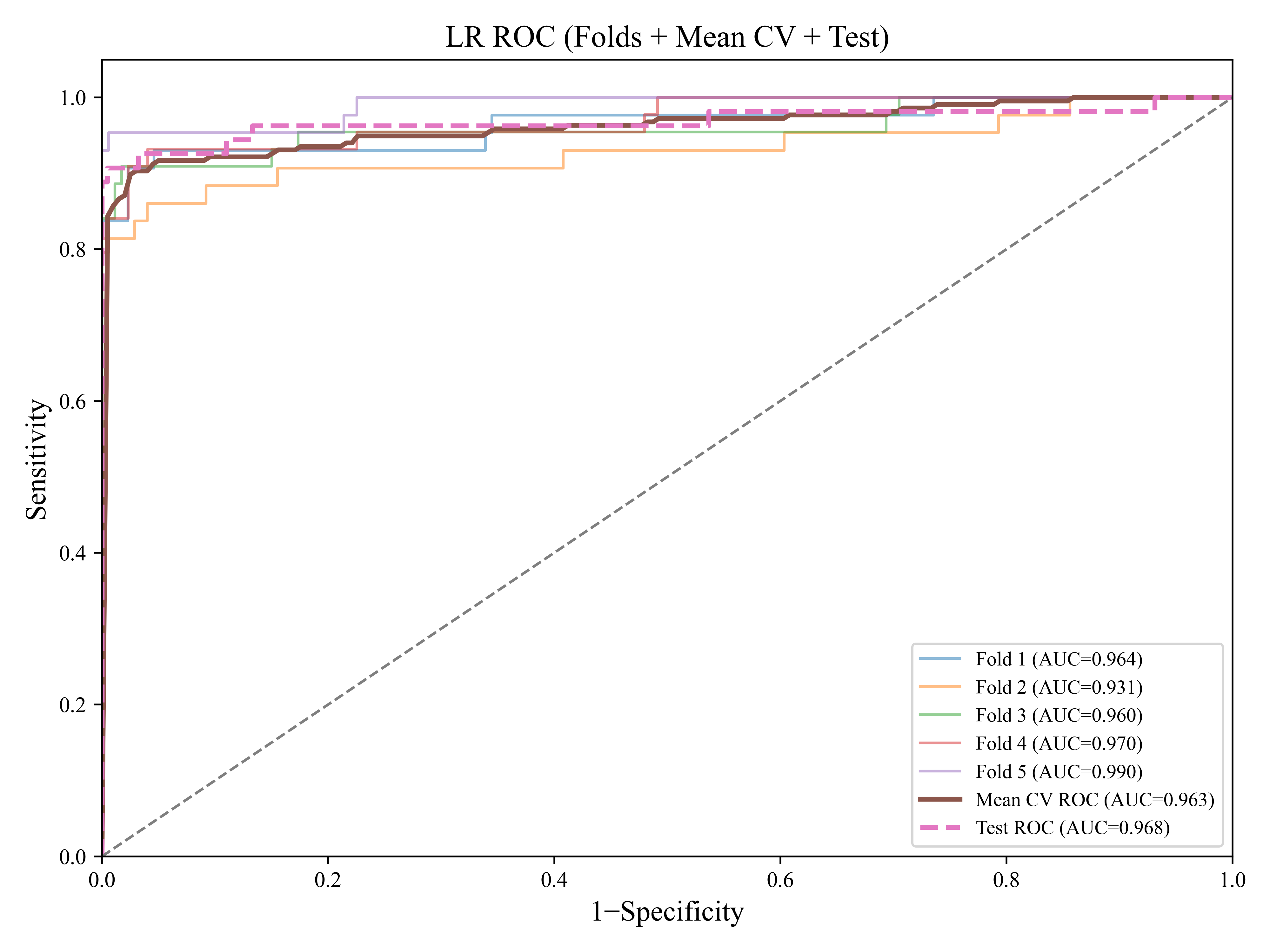


**(e)**


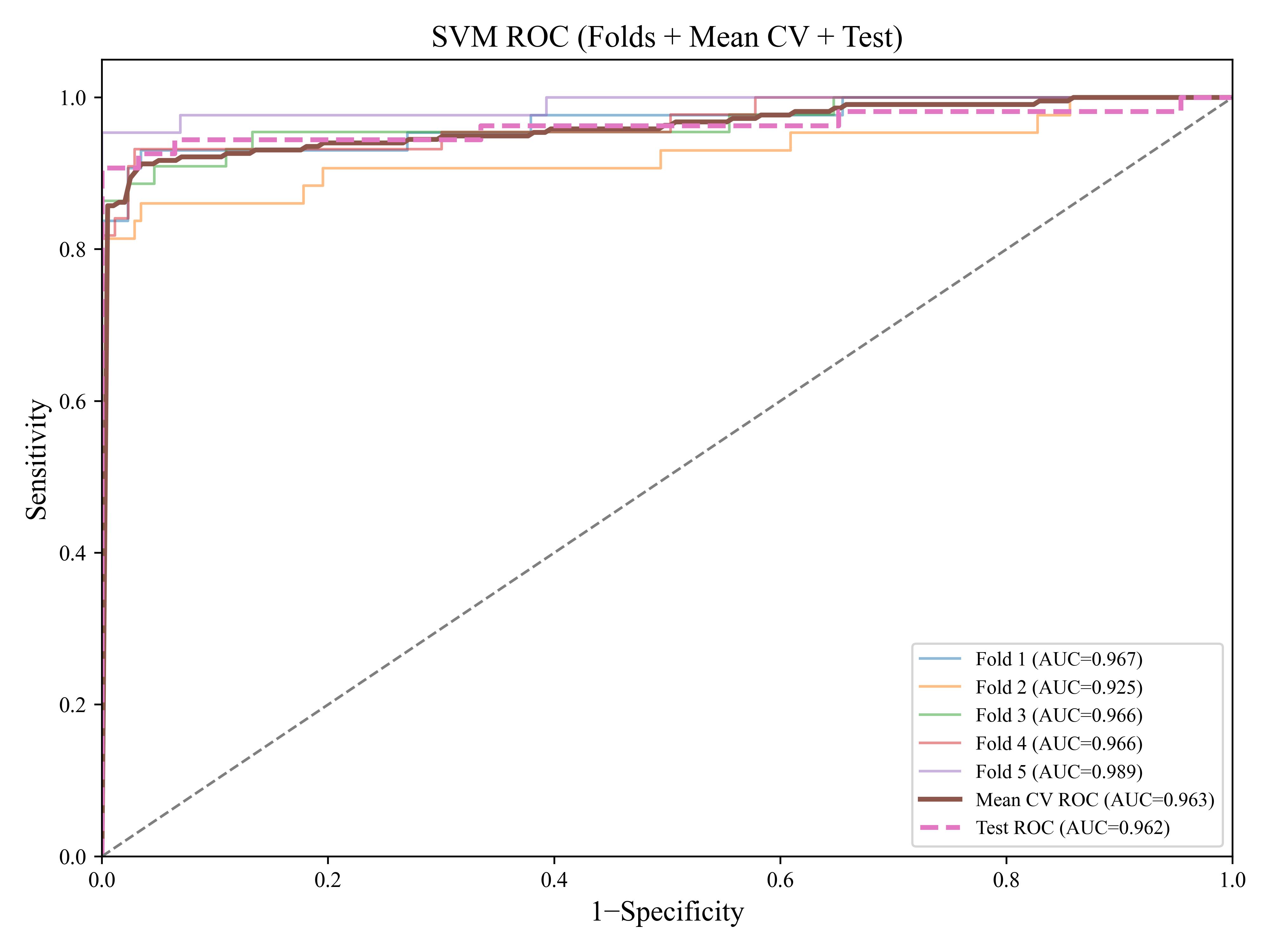


**(f)**

**Figure S5.** SHAP dependency graph (a) LightGBM-Optuna_Age, (b) CatBoost-Optuna_Age, (c) LightGBM-Optuna_FEV1/FVC, (d) CatBoost-Optuna_FEV1/FVC, (e) LightGBM-Optuna_PLT, (f) CatBoost-Optuna_PLT. FEV1/FVC: forced expiratory volume/forced vital capacity; PLT: platelet count.


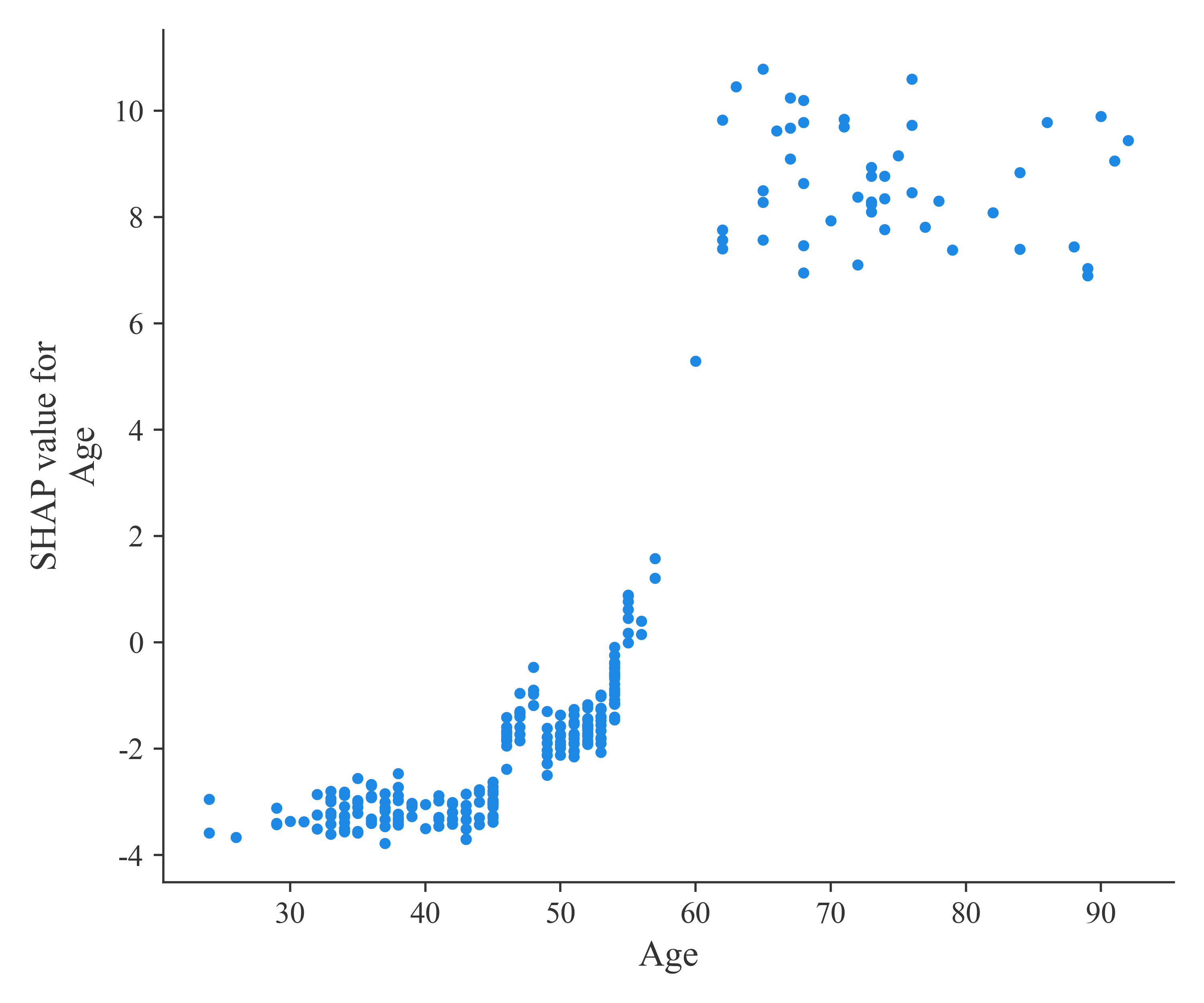


**(a)**


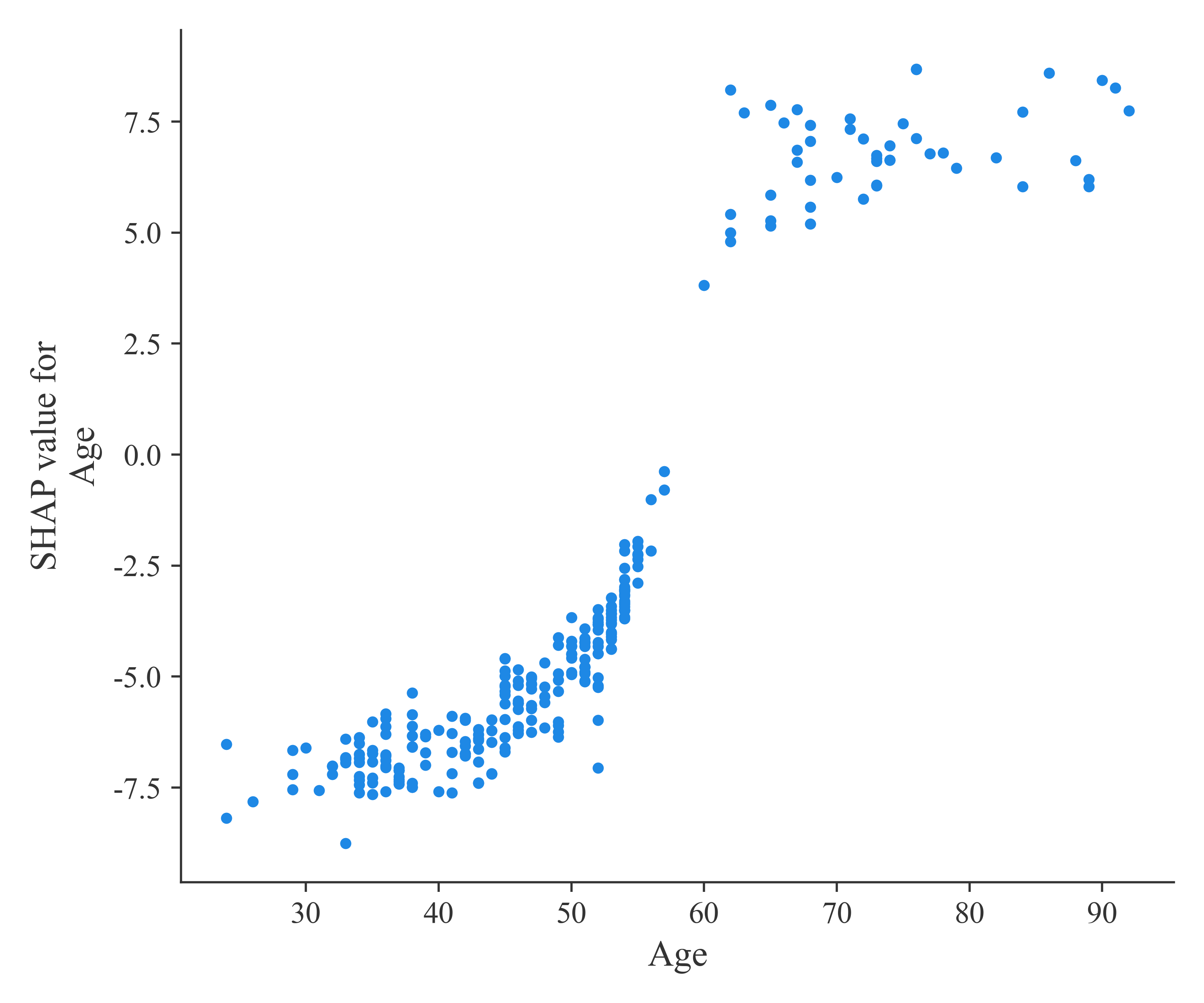


**(b)**


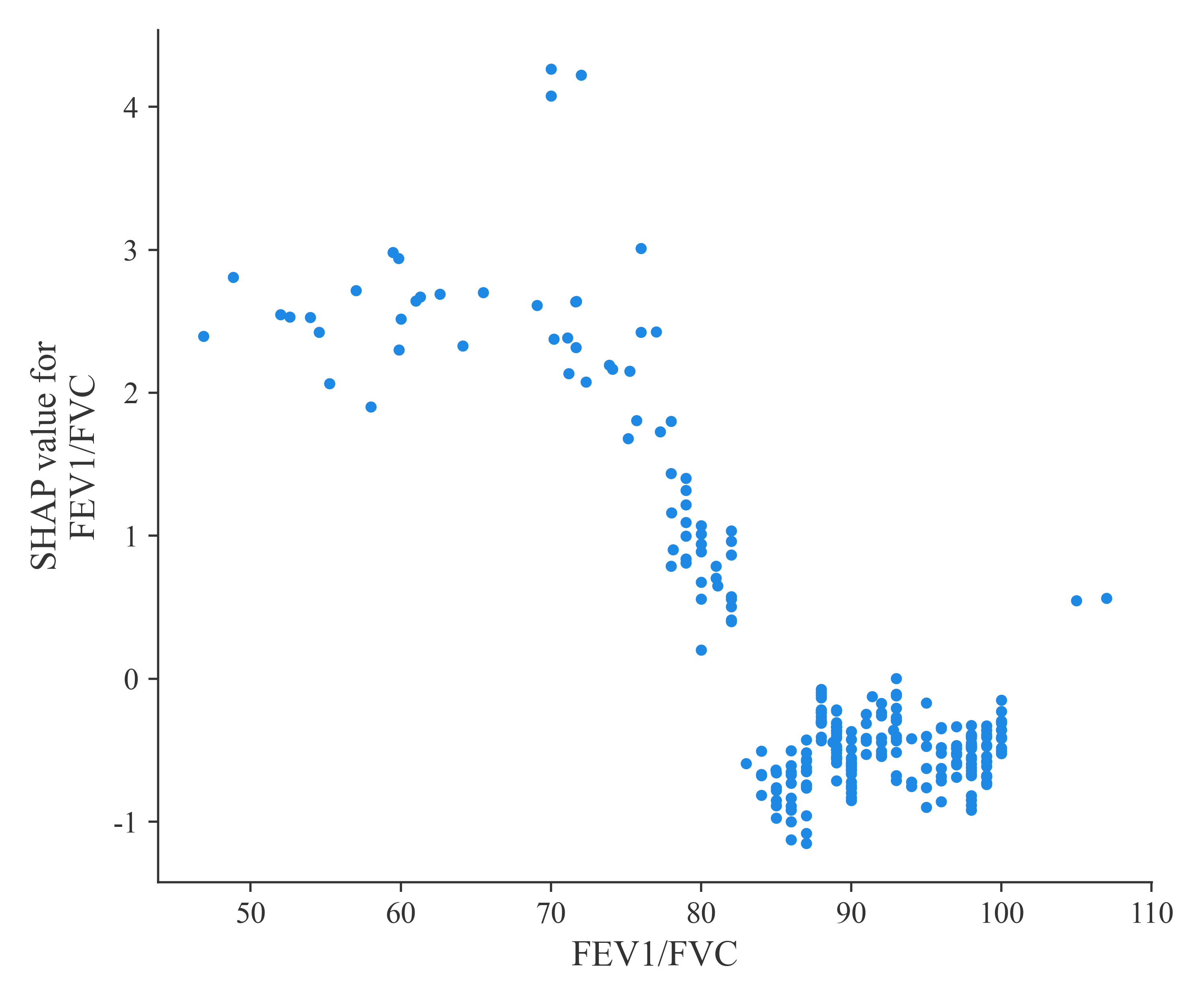


**(c)**


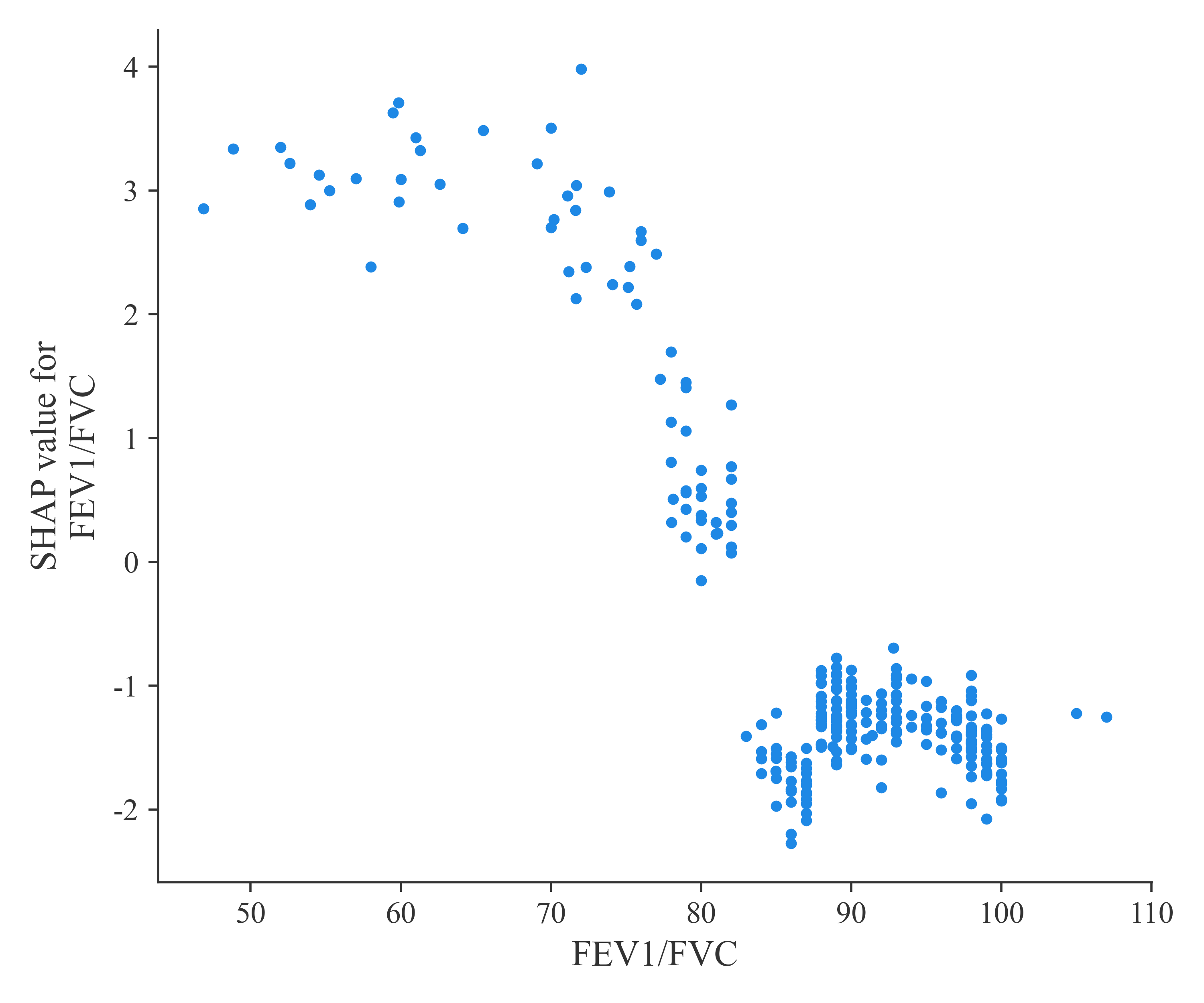


**(d)**


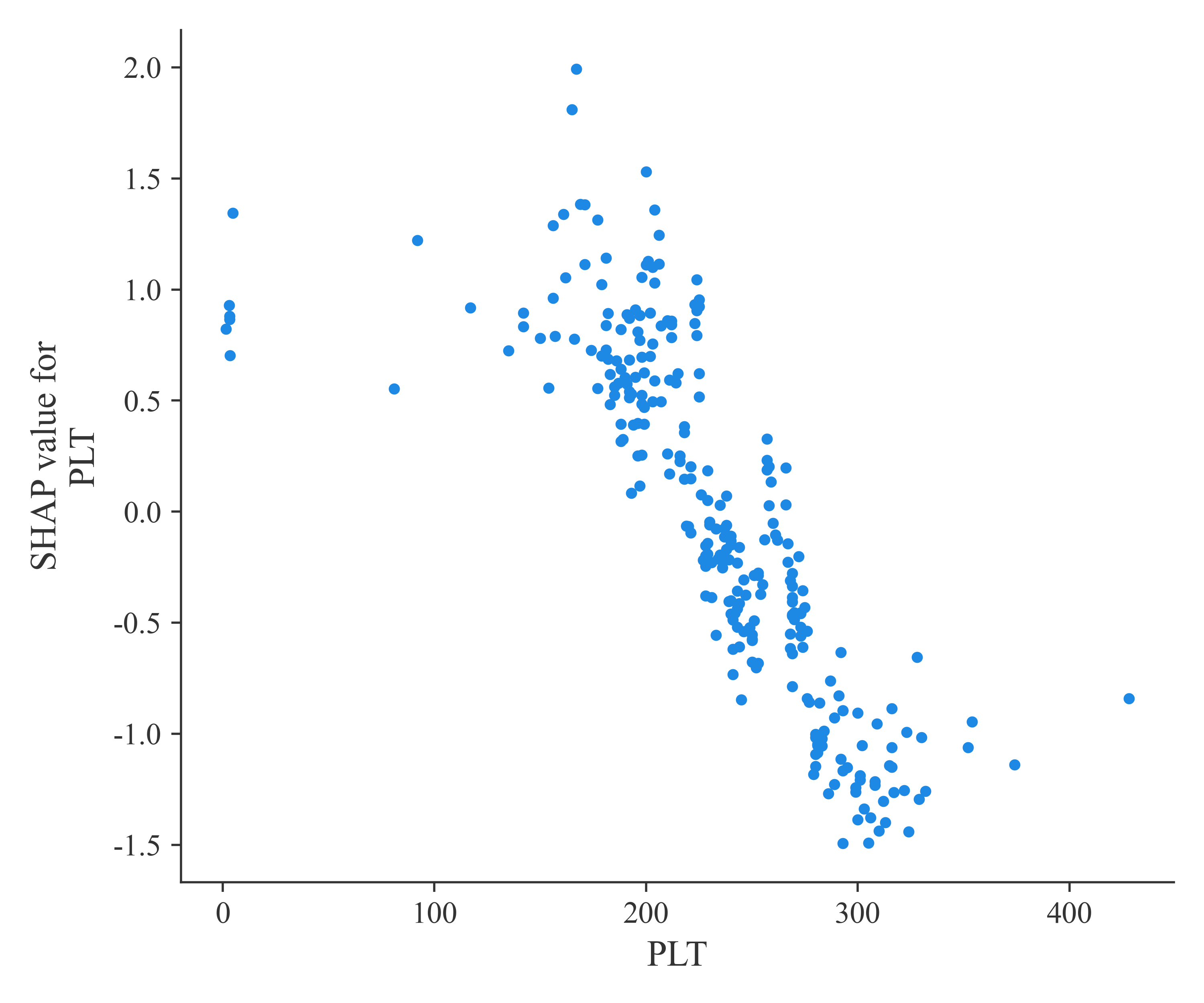


**(e)**


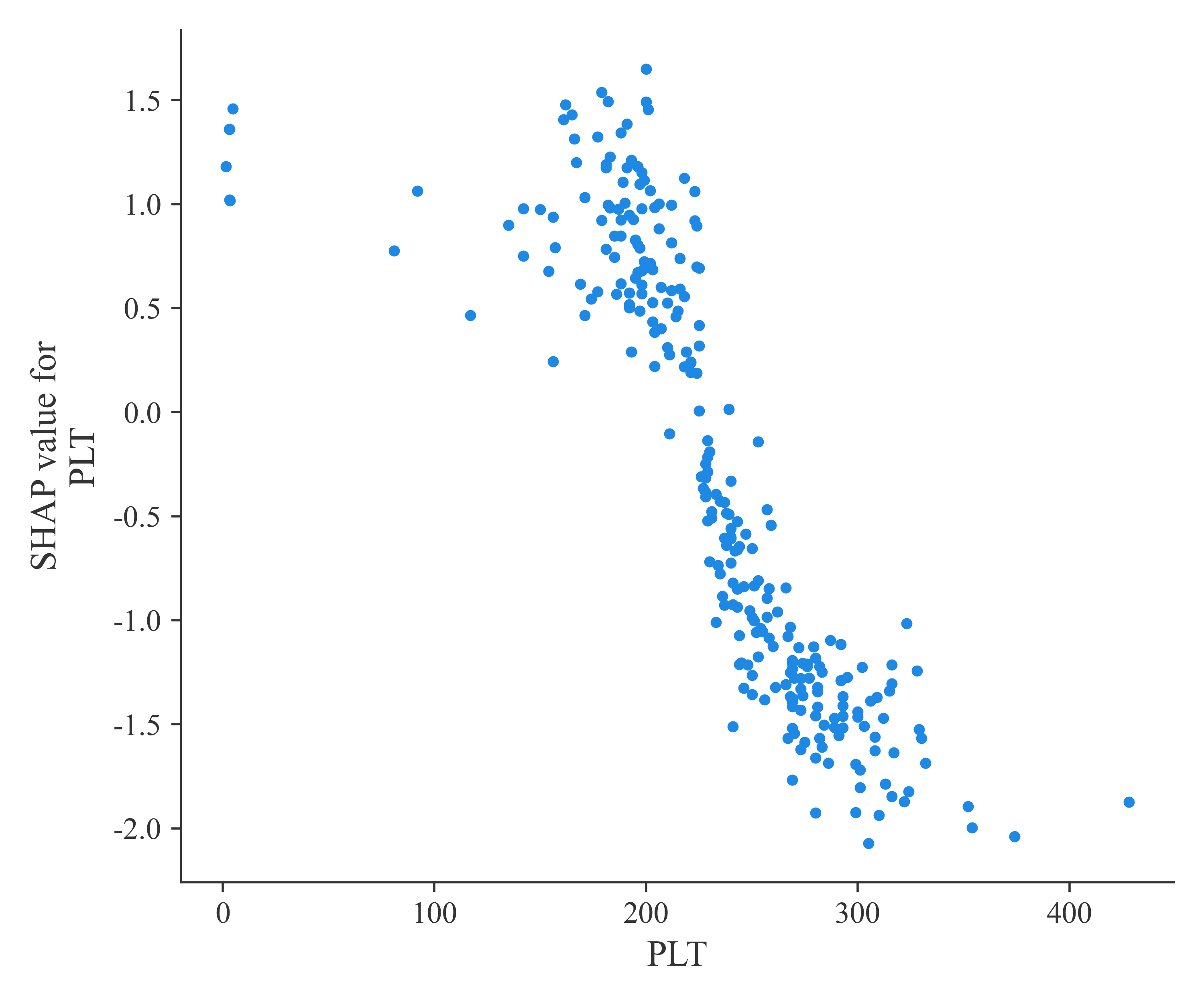


**(f)**
